# Geographical Origin Drives Metabolic Divergence in *Styphnolobium japonicum* cv. Jinhuai: A Widely Targeted Metabolomic Study of Flower Buds from Sichuan and Guangxi, China

**DOI:** 10.3390/metabo16070475

**Published:** 2026-07-07

**Authors:** Leilei Zuo, Yan Chen, Yuxuan Luo, Huan Yang, Dayi Chen, Ying Zhang, Xiao Meng, Waralee Watcharin

**Affiliations:** 1 Modern College of Traditional Chinese Medicine Industry, Chengdu University of Traditional Chinese Medicine, Chengdu 611137, China; zuoleilei@cdutcm.edu.cn; 2 Theophane Venard School of Food Biotechnology and Innovation, Assumption University, Bangkok 10240, Thailand; 3 College of Public Health, Chengdu University of Traditional Chinese Medicine, Chengdu 611137, China; chenyan@cdutcm.edu.cn (Y.C.); 17883461513@163.com (Y.L.); 19160274285@163.com (H.Y.); chendayi00523@sina.com (D.C.); zhangying@cdutcm.edu.cn (Y.Z.)

**Keywords:** *Styphnolobium japonicum* cv. Jinhuai, metabolic divergence, key active ingredient of Chinese medicine, KEGG enrichment pathway

## Abstract

**Highlights:**

**What are the main findings?**

- We identified 1550 metabolites (flavonoid-dominant), 152 key active ingredients of traditional Chinese medicine (TCM-KAIs), and 204 pharmaceutical disease-resistant ingredients (PDRIs) in the flower bud of *Styphnolobium japonicum* cv. Jinhuai (FBSJvJ).
- Geographic origin drove metabolic divergence. Guangxi samples were enriched in lipids and nucleotides, while Sichuan samples were enriched in flavonoids and phenolic acids. In addition, three core biomarkers were identified.

**What are the implications of the main findings?**

- Biomarkers enable origin authentication and precision quality control compared to a single marker (rutin), supporting traceability and regulatory compliance for food and medicinal products.
- Regional metabolic signatures can help identify functional ingredients and guide product formulation, processing optimization, and cultivar selection for food and medicinal development.

**Abstract:**

Background/Objectives: *Styphnolobium japonicum* cv. Jinhuai (SJvJ) represents a medicinal and edible plant whose metabolite composition is strongly shaped by its growing location. Current quality control methods mainly rely on rutin quantification, lacking comprehensive metabolic markers for origin discrimination. Therefore, this study aimed to profile interregional metabolic differences between Guangxi and Sichuan SJvJ flower buds, identify characteristic differential markers, and clarify relevant metabolic pathways, thereby guiding quality control, germplasm evaluation, and functional food development. Methods: Ultra-high performance liquid chromatography–tandem mass spectrometry (UPLC-MS/MS) was employed to identify metabolites. The Traditional Chinese Medicine Systems Pharmacology Database and Analysis Platform (TCMSP) and Cancer HSP were applied to screen the key active ingredients of traditional Chinese medicine (TCM-KAIs) and disease-related pharmaceutical ingredients (PDRIs). Specifically, we targeted six highly prevalent human diseases and another five disorders based on therapeutic indications documented in the Chinese Pharmacopoeia. Multivariate analyses, such as principal component analysis, hierarchical clustering analysis, and other statistical methods, were applied to investigate differential metabolites. The Kyoto Encyclopedia of Genes and Genomes (KEGG) database was utilized for pathway enrichment analysis of marker metabolites. Results: In total, 1550 metabolites were identified across 12 categories, predominantly flavonoids. Additionally, 152 TCM-KAIs and 204 PDRIs against 11 diseases were screened. Multivariate analyses indicated that geographical origin was closely associated with observed metabolic variation among the tested samples: Guangxi samples accumulated higher lipids and nucleotides, whereas Sichuan samples showed higher levels of flavonoids and phenolic acids. Vanilloloside, protocatechuic acid-4-O-glucoside, and gallic acid-4-O-glucoside were identified as key inter-group biomarkers. KEGG enrichment analysis revealed enhanced metabolism of nucleotide/pyrimidine in Guangxi, whereas zeatin biosynthesis was upregulated in Sichuan, consistent with discrepancies in regional climatic patterns. Conclusions: This study established a more comprehensive metabolomic dataset for FBSJvJ. It also clarified the correlations between origin and quality and unraveled the underlying mechanisms. These findings facilitate origin authentication, standardized quality control, and rational exploitation of FBSJvJ as raw materials of functional foods.

## 1. Introduction

Medicinal and edible homologous plants have emerged as research hotspots in modern pharmacology and functional food science. They are valued for their safety, accessibility, and multi-target therapeutic potential, addressing the growing demand for natural health products [1]. Developed over the past three decades, *Styphnolobium japonicum* cv. Jinhuai (SJvJ) is an elite cultivar of *S. japonicum* (L.) Schott. (SJ) specially bred for high-quality flower bud production. It possesses high-quality dried flower buds [2]. This species was formerly placed within the genus *Sophora*, with the synonym *Sophora japonica* L. still adopted as the official medicinal name specified in the Pharmacopoeia of the People’s Republic of China (Chinese Pharmacopoeia, ChP). Taxonomic revisions recorded in Flora of China reclassify this taxon into the genus *Styphnolobium*. The flower buds of SJ (FBSJ), a traditional Chinese medicine (TCM), have long been used clinically for maintaining hemostasis and treating heat-related blood disorders. These flowers are characterized by a bitter, slightly cold nature, and according to the TCM theory, these flowers are associated with the liver and large intestine meridians [3]. Based on the TCM theory, a bitter taste is commonly linked to cold medicinal property, and compounds contributing to bitter flavor, such as flavonoids and alkaloids, are the material basis for the meridian tropism of SJ [4,5].

A key advantage of the flower bud of SJvJ (FBSJvJ) over traditional *S. japonicum* flower bud is the significantly higher content of flavonoids, a critical indicator of medicinal quality, in FBSJvJ. Studies have indicated that the total flavonoid content in FBSJvJ can exceed 40%, with samples from Quanzhou, Guangxi province, reaching over 45% flavonoid content [6]. In contrast, the content of rutin, a major flavonoid component, in traditional FBSJ was reported to range from 13.88% to 29.73% across different producing regions [7]. The ChP sets a minimum rutin standard of 15% for FBSJ [3]. The substantial increase in flavonoid accumulation underscores the enhanced pharmacological potential of FBSJvJ and highlights its relevance for high-value applications in TCM and functional foods. Recent studies have revealed the diverse bioactivities of FBSJ, including anti-inflammatory, antioxidant, and hypolipidemic effects. These properties have been strongly linked to its abundant secondary metabolites, such as flavonoids, terpenoids, and alkaloids [8,9,10,11]. Currently, cancer/tumors, diabetes mellitus, hypertension, cardiovascular diseases, atherosclerosis, and thrombotic diseases are the major diseases threatening human health worldwide [12]. Among them, cardiovascular diseases have surpassed cancer and currently rank first in terms of incidence and mortality [13]. Survey and analysis data have indicated that in recent years, cardiovascular diseases account for approximately 40% of deaths among the Chinese population [14]. Six globally prevalent diseases, including diabetes, cardiovascular diseases, cancer, hypertension, atherosclerosis, and thrombotic disorders, and five additional diseases identified based on therapeutic indications recorded in the ChP(osteoporosis, hepatic ischemic injury, inflammation, infectious diseases, hemorrhage) were set as target diseases for disease-related pharmaceutical ingredient (PDRI) screening.

Metabolomics, particularly widely targeted metabolomics based on ultra-high performance liquid chromatography–tandem mass spectrometry (UPLC-MS/MS), has revolutionized TCM research by enabling systematic profiling of metabolites and deciphering the “multi-component, multi-target, multi-pathway” mechanisms of natural products [15]. This approach integrates the broad coverage of untargeted metabolomics with the high accuracy and precision of targeted methods, making it well-suited for analyzing complex matrices, such as TCM [16]. In combination with the multi-reaction monitoring (MRM) mode of triple-quadrupole (QQQ) mass spectrometry, it allows precise qualitative and quantitative detection of trace metabolites and contributes to the identification of potential active components and geographic variations [17].

Regarding chemical composition and metabolic characteristics, UPLC-MS/MS was applied to investigate the metabolic changes of SJ, indicating that the metabolites of this plant undergo significant dynamic changes during flower maturation. Among these metabolites, flavonoids and phenolic acids were identified as the core differential components, which directly affect the quality of the plant [18]. In another study on SJvJ, endogenous hormone levels during flower bud differentiation exhibited the following order: ABA (abscisic acid) > IAA (indole acetic acid) > ZR (zeatin riboside) > GA_3_ (gibberellin). A lower hormone ratio was found to facilitate the transition from vegetative to reproductive growth, providing insight into the flowering mechanism of this variety [19].

By integrating widely targeted metabolomics with network pharmacology and molecular docking, the protective effect of FBSJvJ against alcohol-induced liver injury was predicted. Furthermore, specific flavonoid glycosides with the highest potential as hepatoprotective metabolites were detected [20]. Meanwhile, a preliminary study on the metabolic differences of FBSJ from different producing areas revealed that geographic origin features one of the key factors shaping the metabolite profile of FBSJ. For quality evaluation and standardization, a grading system based on rutin content was proposed for SJvJ from Guangxi, identifying seedlings aged 2–3 decades and the SJvJ J3 variety as optimal quality [21]. Another study integrated metabolomics with physicochemical indicators to establish a quality grading framework for FBSJvJ. The study incorporated measures such as purity, thousand-grain weight, and rutin content (≥33.73% for Grade I), providing a reference for quality control [6]. However, despite the above-mentioned progress in FBSJvJ research, there are still many directions that need further exploration: (1) Previous studies have focused on FBSJvJ flavonoids (e.g., rutin) and liver protection, but they did not systematically detect overall metabolites, key TCM components, or metabolites targeting diseases, like cancer and diabetes [6,20]. (2) Sichuan (Dazhu) and Guangxi are two important FBSJvJ-producing areas, but there is no systematic comparison of their metabolite profiles, origin-specific markers, or marker-related pathways. (3) Current quality standards, which rely on rutin and physicochemical indicators, lack metabolomic-based markers for reliable authentication of origin.

To address these gaps, we applied our established widely targeted UPLC-MS/MS metabolomics platform to analyze three FBSJvJ groups from Dazhu (Sichuan) and one native group from Quanzhou (Guangxi) [20]. Quanzhou (Guangxi), regarded as the core production area of cultivar, and Dazhu, an important introduced cultivation base, were chosen to capture cultivar- and introduction-related metabolic variations. The results are intended to guide regional cultivation strategies and industrial development of FBSJvJ. The objectives of this study were to (1) expand metabolome coverage and identify key active components of TCM and disease-related metabolites via the TCMSP database; (2) compare differences in metabolites between FBSJvJ obtained from Sichuan and Guangxi using multivariate statistics, including principal component analysis (PCA) and hierarchical cluster analysis (HCA); (3) screen origin-specific marker metabolites; and (4) investigate biosynthetic pathways of these markers through The Kyoto Encyclopedia of Genes and Genomes (KEGG) annotation and pathway enrichment analysis, thereby elucidating the molecular basis underlying geographic metabolic variation.

This study expands the FBSJvJ metabolomic database, identifies understudied components, such as phenolic acids and nucleotides, and delineates Sichuan–Guangxi metabolic signatures. These findings support precise origin authentication, quality optimization, and the development of FBSJvJ as a promising raw material for functional foods, thereby contributing to the utilization of SJvJ resources.

## 2. Materials and Methods

### 2.1. Materials

Chromatographic grade methanol and acetonitrile were purchased from Shanghai Merck Chemical Technology Co., Ltd. (Shanghai, China), while chromatographic grade formic acid was purchased from Shanghai Aladdin Biochemical Technology Co., Ltd. (Shanghai, China). FBSJvJ samples were collected from two geographic origins in China: SJvJgx from Guangxi (110°37′~111°29′ E, 25°29′~26°23′ N, Quanzhou), sourced from Guilin Yuanxinda Biotechnology Co., Ltd. Development Co., Ltd. (Guilin, China); SJvJsc (including three selected elite lines: SJvJsc1, SJvJsc2, SJvJsc3) from Sichuan (106°59′~107°32′ E, 30°20′~31°00′ N, Dazhu), obtained from Sichuan Huaijin Agricultural Development Co., Ltd. (Dazhou, China). All samples were harvested in July 2022 and were identified by Assistant Professor Jihai Gao from Chengdu University of TCM in accordance with morphological criteria specified in the ChP. Voucher specimens of these materials were deposited in the State Bank of Chinese Drug Germplasm Resources (Voucher numbers: 522230190125HM005, 522230190125HM006, 522230190125HM007, and 522230190125HM008).

### 2.2. Sample Preparation and Extraction

A 50 mg freeze-dried powder of FBSJvJ was dissolved in 1.2 mL of pre-cooled (−20 °C) 70% methanol. The mixture was vortexed for 30 s every 30 min, and vortexing was repeated six times. The sample was subsequently centrifuged at 1200 rpm for 3 min at 4 °C, and the supernatant was filtered through a microporous membrane for subsequent UPLC-MS/MS analysis. Furthermore, 70% methanol-water was employed as the blank group.

### 2.3. UPLC-MS/MS Analysis

Metabolite identification was conducted using a UPLC-MS/MS system comprising an ExionLC™ AD UPLC and a 4500 Q TRAP MS (AB Sciex, Marsiling, Singapore).

#### 2.3.1. Liquid-Phase Conditions

Data acquisition was conducted using a UPLC system coupled with MS/MS. The liquid phase conditions included an Agilent SB-C18 column (1.8 µm, 2.1 mm × 100 mm). Operational conditions were as follows: column temperature: 40 °C; mobile phase: solvent A (0.1% formic acid in water) and solvent B (0.1% formic acid in acetonitrile); flow rate: 0.35 mL/min; and injection volume: 4 μL. Gradients: 0–9.0 min, 5–95% B; 9.0–10.0 min, 95–95% B; 10.0–11.1 min, 95–5% B; 11.1–14.0 min, 5–5% B.

#### 2.3.2. MS Conditions

The effluent was analyzed using linear ion trap and QQQ scans. Electrospray ionization temperature was set to 550 °C, with an ion spray voltage of 5500 V (positive ion mode) or −4500 V (negative ion mode). The ion source gas was set to 50 psi, gas II was set to 60 psi, and the curtain gas was set to 25 psi. Collision-induced ionization parameters were set to “high”. The QQQ scan was conducted using the MRM mode, with the collision gas (nitrogen) set to medium. The declustering potential and collision energy were optimized for each MRM ion pair. A specific set of MRM ion pairs was monitored during each period based on the metabolites eluted during that time.

### 2.4. Qualitative Determination of Metabolites

For qualitative analysis of metabolites, the data analysis method was adapted from the widely targeted metabolomics qualitative workflow [22].

The substances were identified based on fragmentation patterns, retention time, the Metware database (MWDB) V2.0, and a public database established based on *m*/*z* values provided by Metware Biotechnology Co., Ltd. (Wuhan, China). Using secondary mass spectra information, the obtained metabolite data were compared with the MWDB database to obtain structural information and classifications. Thereafter, differential metabolites between FBSJvJ samples were studied. Isotopic signals, repetitive signals containing K^+^, Na^+^, and NH_4_^+^, and fragment ions derived from larger molecular species were excluded before analysis.

Quantitative analysis was conducted using the MRM mode of QQQ mass spectrometry. After obtaining the mass spectrometry data for different samples, the peak area normalization method was applied to calculate the relative abundance of FBSJvJ samples from different producing areas. Each sample was analyzed in triplicate, and the average value was calculated [23].

### 2.5. Identification of Key Active Ingredients in Traditional Chinese Medicine (TCM-KAIs)

UPLC-MS/MS was employed to analyze FBSJvJ, and all metabolites were queried in the TCMSP database. Metabolites with oral bioavailability (OB) ≥ 5% and drug-likeness (DL) ≥ 0.14 were identified as TCM-KAIs. The relevant targets of the identified metabolites, along with associated disease information, were obtained [24].

### 2.6. Identification of PDRIs

Based on the clinical importance of diseases and their reported efficacy, the disease spectrum for the study was determined using the TCMSP database (https://www.tcmsp-e.com/#/database, accessed on 2 July 2026) and the CancerHSP database. PDRIs associated with multiple disease categories included six major core diseases (CDs), comprising diabetes, cardiovascular diseases, cancer, hypertension, atherosclerosis, and thrombotic disorders, as well as five additional conditions associated with efficacy indications described in the ChP (ChP-related diseases), namely osteoporosis, hepatic ischemic injury, inflammation, infectious diseases, and hemorrhage. Finally, by comparing the metabolites identified through UPLC-MS/MS analysis with disease-resistant components, PDRIs in FBSJvJ were determined [25].

### 2.7. MS Data Analysis and Analysis of Metabolic Mechanisms

Using relevant software, PCA, HCA, and orthogonal partial least squares discriminant analysis (OPLS-DA) were conducted on the identified metabolites of flower buds from SJvJ. Analysis tools, such as Venn diagrams and box plots, were used to localize the sources of differences among SJvJgx and SJvJsc flower buds and identify the marker metabolites. Differential metabolites were selected based on variable importance in projection (VIP) obtained from the OPLS-DA model, considering VIP ≥ 1 and fold change (FC) ≥ 2 or FC ≤ 0.5 as the selection criteria.

### 2.8. KEGG Annotation and Enrichment Analysis

Using the KEGG database, related metabolic pathways were screened for metabolites from the four groups of FBSJvJ with different origins [26]. Using the OPLS-DA model, differential metabolites were identified based on two criteria: VIP ≥ 1 and *p*-value < 0.05 (ANOVA). These differential metabolites were aligned and annotated using the KEGG metabolic library and then mapped to the KEGG Pathway database [27]. Further enrichment analysis of the mapped pathways was conducted using the network-based server Metabolite Sets Enrichment Analysis (MSEA). Pathways with *p* ≤ 0.05 were considered significantly enriched [25].

### 2.9. Data Analysis

All statistical analyses and data visualizations were conducted using R software. PCA was conducted using the base package (version 3.5.1), and data were pretreated by unit variance (UV) scaling. Heatmap visualization was conducted using the ComplexHeatmap package (version 2.8.0), where data were also subjected to UV scaling. OPLS-DA S-plots were constructed employing the corrplot package (version 0.84), without additional data preprocessing. OPLS-DA was implemented using the MetaboAnalystR package (version 1.0.1), for which data were processed using log2 transformation and centralization before analysis.

## 3. Results

### 3.1. Overall Analysis of the Widely Targeted Metabolites of FBSJvJ

**Figure 1:**
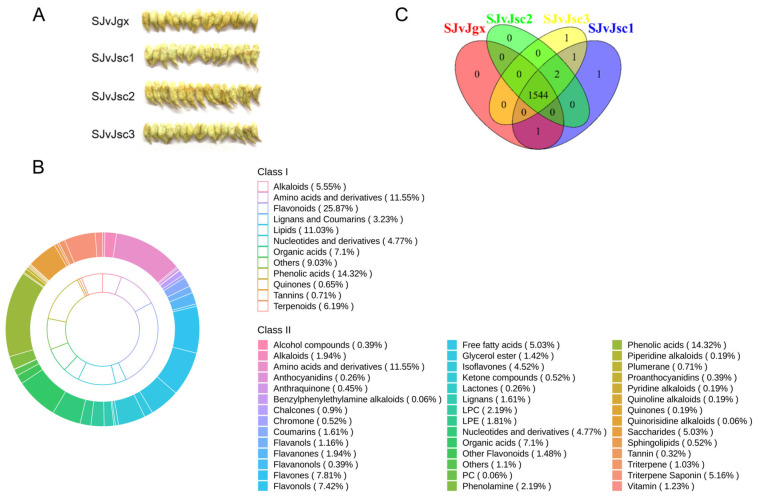
Flower buds of *Styphnolobium japonicum* cv. Jinhuai from Guangxi (SJvJgx) and Sichuan (SJvJsc) (**A**), ring chart representation of FBSJvJ metabolite profiles (**B**), and Venn diagram of shared and unique metabolites from the four groups of FBSJvJ (**C**).

According to the ChP [3], FBSJ is typically oval to elliptical, 2–6 mm long, and approximately 2 mm in diameter. The lower part of the calyx shows several longitudinal ribs, above which unopened, yellow-white petals can be found. Pedicels are slender. The material is light and friable when pinched, with a faint odor and a mildly bitter-astringent taste. All FBSJvJ samples in this study conformed to these diagnostic features but exhibited distinctive, cultivar-linked morphology. The specimens were generally spindle-shaped (fusiform), tapering to an acuminate apex, a feature typically observed in immature floral buds. They were uniformly pale yellow in terms of color, consistent with physiological freshness and lack of senescent pigmentation. In each sample group, the specimens showed relative homogeneity in terms of bud length. Figure 1A shows the shape, color, and relative size of FBSJvJ from Guangxi and Sichuan. Four consistent morphological differences were observed between groups. The Guangxi group (SJvJgx) was relatively slender and short, with the calyx largely enclosing the corolla, and calyx lobes remaining closed. The Sichuan group 1 (SJvJsc1) was fusiform with a swollen middle and medium length, exhibiting mostly full calyx enclosure. The Sichuan group 2 (SJvJsc2) comprised the most elongated and longest buds, where the calyx typically covered only the lower two-thirds of the bud, and calyx lobes were often half-open. The Sichuan group 3 (SJvJsc3) was stout and the shortest, with a fully enclosing calyx and closed lobes. Across groups, pedicels were consistently fine, and buds were lightweight and easily crushed, with a faint smell and a slightly bitter-astringent taste.

A UPLC-MS/MS-based metabolomic analysis was conducted on four types of FBSJvJ. In total, 1550 metabolites were identified across all tested samples (Figure 1B), comprising 401 flavonoids, 171 lipids, 222 phenolic acids, 86 alkaloids, 74 nucleotides and their derivatives, 179 amino acids and their derivatives, 10 quinones, 50 lignans and coumarins, 11 tannins, 96 terpenoids, 110 organic acids, and 140 other types of metabolites.

Sub-class profiling revealed that flavonoids predominantly consisted of flavones (121), flavonols (115), and isoflavones (70), whereas anthocyanins were the least abundant metabolites (4). Within other classes of metabolites, triterpenoid saponins were the most prevalent terpenoids (80), free fatty acids represented the largest proportion of lipids (78), saccharides constituted 78 metabolites, and alkaloids were primarily composed of phenolamines (35) and alkaloid bases (30).

A Venn diagram analysis was performed for the four groups of FBSJvJ samples (SJvJgx, SJvJsc1, SJvJsc2, SJvJsc3) to more intuitively illustrate the differences in metabolite categories among samples from different origins (Figure 1C). In total, 1544 metabolites were shared across all four groups. Two metabolites, namely 8-C-glucosyl-noreugenin and Lysophosphatidylcholine 19:3 (LysoPC 19:3), were unique to the Sichuan-grown samples (SJvJsc1, SJvJsc2, SJvJsc3) compared with the Guangxi-grown samples (SJvJgx). In addition, SJvJsc1 contained one exclusive metabolite, 2,3-dihydroxyurs-12-en-28-oic acid methyl ester (corosolic acid methyl ester), while SJvJsc3 harbored one unique metabolite, L-histidine-L-threonine-L-lysine-L-lysine (His-Thr-Lys-Lys). Among the shared subsets, soyasaponin H glucuronic acid glucose rhamnoside was detected exclusively in SJvJsc1 and SJvJsc3, whereas ethyl gallate was the only metabolite shared exclusively between SJvJsc1 and SJvJgx.

### 3.2. Identification of TCM-KAIs from FBSJvJ

To characterize the pharmacological components of FBSJvJ, we screened for TCM-KAIs using the TCMSP database. All detected metabolites were cross-referenced against the TCMSP database. Among the 1550 metabolites identified in this study, 320 were included in the TCMSP database. Furthermore, 151 potential TCM-KAIs were selected considering the thresholds of OB ≥ 5% and DL ≥ 0.14. Although rutin (OB = 3.2%, DL = 0.68) did not meet this screening criterion, it is a known agent with health-promoting properties; therefore, it was also included as TCM-KAI (Table 1). Thus, 152 TCM-KAIs were identified, and flavonoids constituted the most abundant class, comprising 97 compounds (~63% of all TCM-KAIs). However, corosolic acid methyl ester was exclusively detected in the SJvJsc1 sample.

In total, 113 of these 152 TCM-KAIs were predicted to interact with 358 target proteins. These targets were associated with 351 distinct diseases. These diseases primarily encompassed cancer, cardiovascular disorders, inflammatory conditions, Alzheimer’s disease, osteoporosis, metabolic syndrome, skin diseases, diabetes, and obesity. This target-disease network suggests that FBSJvJ may possess preventive or therapeutic potential against these conditions.

Notably, 17 of the 152 identified TCM-KAIs exhibited high DL (DL > 0.65) despite lacking associated protein target data. Their favorable molecular properties indicated significant bioactivity, representing promising candidates for future mechanistic studies and potential drug development. These 17 metabolites were as follows: apigenin, 6″-O-malonylglycitin, kaempferol-7-O-rhamnoside, xanthohumol, eriodictyol-7-O-glucoside, biochanin A-7-O-glucoside (sissotrin), 6″-O-malonyldaidzin, 6″-O-acetyldaidzin, conazol H, acacetin-7-O-rutinoside (linarin), olivil-4′-O-glucoside, olean-12-ene-3,22,23-triol (soyasapogenol B), 3,23-dihydroxylup-20(29)-en-28-oic acid (23-hydroxybetulinic acid), 3-hydroxyurs-12-en-28-oic acid (ursolic acid), 3,24-dihydroxyolean-12-en-22-one (soyasapogenol E), dehydrosoyasaponin I, and rubiarbonol C.

### 3.3. Identification of Disease-Resistant Active Ingredients of FBSJvJ

Concerning disease-related metabolite screening, from the 319 TCM ingredients identified, 204 metabolites were associated with the 11 above-mentioned diseases in the CancerHSP and TCMSP databases. The 204 metabolites were annotated as PDRIs. In total,186 metabolites in FBSJvJ samples were associated with CDs, among which flavonoids were the most abundant metabolites, accounting for 84 species (Table S1). Additionally, 179 metabolites in FBSJvJ samples were associated with ChP-related diseases, with flavonoids representing the most prevalent metabolites (86 species). However, comparatively fewer metabolites were associated with hemorrhage and liver injury. This discrepancy may be partly due to the relatively limited annotation of compound-disease relationships for these conditions in the TCMSP database. This may have affected the coverage of target prediction results.

### 3.4. Differential Metabolites in FBSJvJ

#### 3.4.1. Cluster Analysis

**Figure 2:**
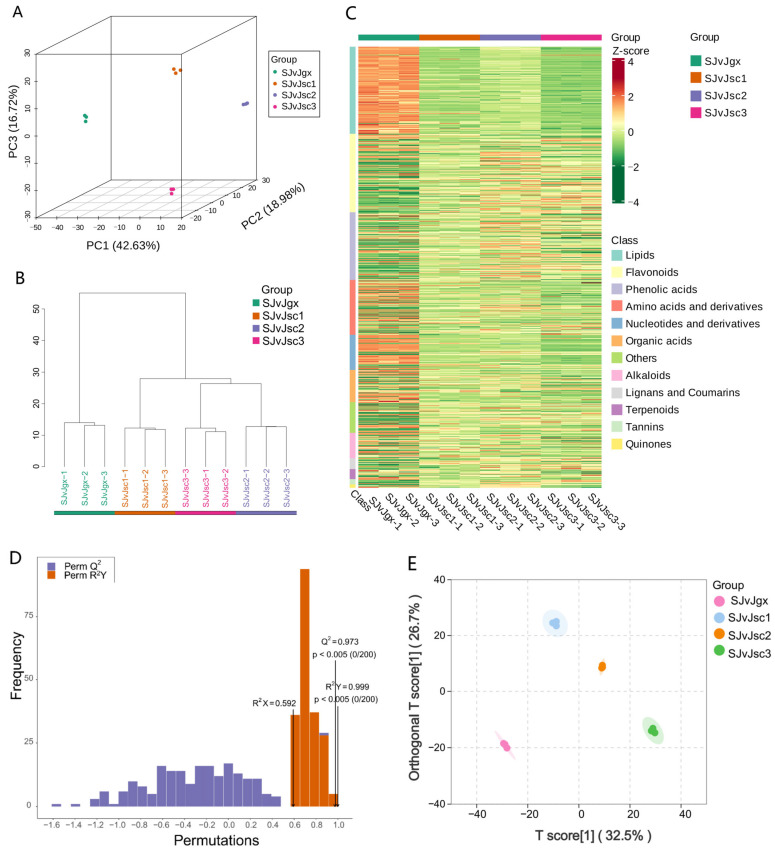
3D PCA plot of the four FBSJvJ samples. 3D PCA plots (**A**), HCA dendrogram (**B**), heatmap visualization of the abundance of differential metabolites, the category class, and panorama (**C**), orthogonal partial least squares discriminant analysis (OPLS-DA) model verification diagrams (**D**), and OPLS-DA score plot of the four FBSJvJ groups (**E**).

We conducted PCA and HCA to assess metabolic variation across FBSJvJ samples. The 3D PCA score plot (Figure 2A) revealed distinct clustering based on sample type and geographic origin. Specifically, the samples formed four separate clusters corresponding to the three FBSJvJsc lines from Sichuan and the one FBSJvJgx group from Guangxi. This finding indicates that metabolite profiles differed more substantially between producing regions than between genetic lines in the same region. Biological replicates within each sample type clustered tightly, suggesting high experimental reproducibility. The first three principal components (PC1–PC3) cumulatively explained over 75% of the total variance, sufficiently capturing the major sources of metabolic variation among the samples.

HCA corroborated the results of PCA, confirming distinct metabolic profiles between FBSJvJgx and FBSJvJsc and more subtle differences among the three breeding lines from Sichuan (SJvJsc1, SJvJsc2, and SJvJsc3) (Figure 2B). The dendrogram topology reflected these relationships. Herein, SJvJsc3 and SJvJsc1, which exhibited the highest metabolic similarity, clustered first, followed by SJvJsc2. In addition, the Guangxi sample (SJvJgx) merged last.

These trends were visualized using a differential metabolite heatmap (Figure 2C). Guangxi samples were characterized by relatively higher abundances of lipids, amino acids and their derivatives, nucleotides and their derivatives, and selected organic acids. In contrast, Sichuan samples were enriched in flavonoids, phenolic acids, and tannins. Together, PCA and HCA indicated that both geographic origin and genetic line within a region significantly modulate the metabolome of FBSJvJ, with inter-regional variation featuring the dominant factor.

#### 3.4.2. OPLS-DA

Unlike unsupervised PCA, supervised OPLS-DA maximizes inter-group separation to more effectively identify discriminatory metabolites. The OPLS-DA algorithm decomposes the predictor matrix (X, metabolite data) into two parts: a predictive component correlated with the class labels (Y, group membership) and an orthogonal component not correlated with Y. By filtering out this orthogonal variation, the model effectively removes systematic noise irrelevant to class distinction, thereby isolating variables that genuinely discriminate between the FBSJvJ groups.

A 200-iteration permutation test was conducted to validate the OPLS-DA model and identify the differential metabolites driving group separation (Figure 2D). In OPLS-DA, the parameters R^2^X and R^2^Y represent the explained variance of the model for the X- and Y-matrices, respectively, while Q^2^ indicates its predictive accuracy.

The model exhibited excellent performance and high predictive capability, characterized by a Q^2^ value of 0.973 (*p* = 0.005). The explanatory power of the model was also robust: R^2^X = 0.592 and R^2^Y = 0.999. This finding indicates a reasonable fit to the metabolite data and an outstanding representation of group separation. Critically, the permutation test confirmed the validity of the model, as the *p*-value for R^2^Y was <0.005. This demonstrates that none of the random grouping models outperformed the actual model in explaining the Y-matrix.

These results collectively validate the OPLS-DA model as an effective tool for distinguishing FBSJvJ groups and provide a reliable basis for subsequent differential metabolite screening.

The OPLS-DA score plot (Figure 2E) revealed distinct metabolic phenotypes among FBSJvJ samples from different geographical origins and cultivars. The first predictive component (T[1]), which captured 32.5% of the total variance, effectively separated the Guangxi sample (SJvJgx) from the three Sichuan cultivars (SJvJsc1, SJvJsc2, and SJvJsc3). The 95% confidence ellipses of Hotelling’s T^2^ exhibited no overlap, confirming that provenance remains the primary factor driving metabolic divergence.

Along the orthogonal component, the three Sichuan cultivars displayed subtle but detectable separation. SJvJsc2 and SJvJsc3 clustered closely together, while SJvJsc1 was relatively isolated, suggesting measurable intra-regional variation in chemotype. All biological replicates fell within their respective confidence ellipses, supporting data reproducibility.

Collectively, the results of OPLS-DA suggest that geographical origin is the dominant factor affecting the metabolomic profiles of FBSJvJ. In addition, cultivar-specific differences within Sichuan were identified as secondary but detectable factors affecting the metabolomic profiles of FBSJvJ.

### 3.5. Screening of Differential Metabolites of FBSJvJ from Different Groups

**Figure 3:**
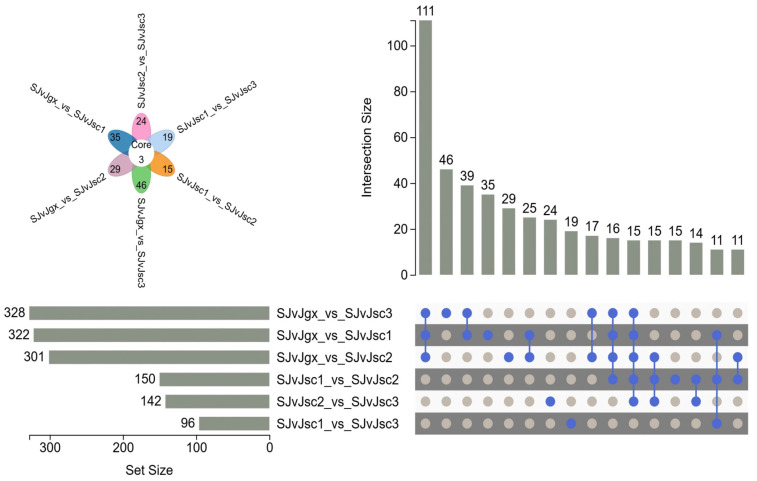
Upset plot embedded with the petal plot of differential metabolites among FBSJvJ from the four groups. Blue dots stand for pairwise comparison groups, linked dots represent group intersections, and the vertical bars above denote the count of shared differential metabolites in each intersection.

First, an OPLS-DA model including all four groups of FBSJvJ (SJvJgx, SJvJsc1, SJvJsc2, and SJvJsc3) was established to screen metabolites across groups. The criteria of VIP ≥ 1, |log2FC| ≥ 1, and *p* ≤ 0.05 in this multigroup model yielded 658 differential metabolites, comprising 117 flavonoids, 130 lipids, 101 phenolic acids, 82 amino acids and conjugates, 52 nucleotides and their derivatives, 48 organic acids, 38 alkaloids, 16 lignans and coumarins, 15 terpenoids, 8 tannins, 5 quinones, and 46 other metabolites. Subsequently, strict pairwise comparisons were conducted for all six group pairs based on the criteria of VIP ≥ 1, |log2FC| ≥ 1, and *p* ≤ 0.05, yielding 572 differential metabolites (Figure 3).

**Figure 4:**
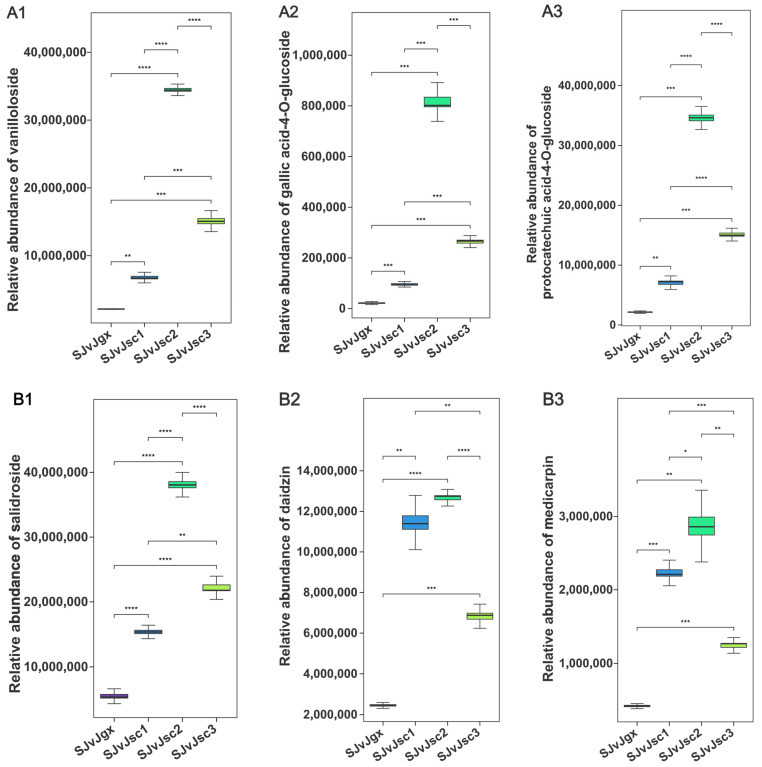
Boxplots of 3 metabolites (**A1**–**A3**) common to all six pairwise comparisons and 3 metabolites (**B1**–**B3**) enriched in the SJvJsc1–3 groups compared to the SJvJgx group of FBSJvJ. Note: Group comparisons were conducted using the *t*-test: * *p* < 0.05, ** *p* < 0.01, *** *p* < 0.001, **** *p* < 0.0001.

Inter-regional contrasts between the Guangxi group and each Sichuan-introduced group produced substantially more differential metabolites (SJvJgx vs. SJvJsc3: 328; vs. SJvJsc1: 322; vs. SJvJsc2: 301) than pairwise comparisons among the three groups from Sichuan (SJvJsc1 vs. SJvJsc2: 150; SJvJsc2 vs. SJvJsc3: 142; SJvJsc1 vs. SJvJsc3: 96), indicating greater metabolic divergence between regions than between the introduced groups from Sichuan. Three metabolites, namely vanilloloside, protocatechuic acid-4-O-glucoside, and gallic acid-4-O-glucoside, were identified as candidate discriminatory biomarkers in all six comparisons (Figure 4A1–A3). These phenolic acid glucosides typically show improved aqueous solubility and physicochemical stability while retaining much of the antioxidant property [28,29,30] of their parent aglycones.

Analysis of unique differentials highlighted this pattern: comparison of SJvJgx with SJvJsc3 exhibited the most unique differential metabolites (*n* = 46), followed by the comparison of SJvJgx with SJvJsc1 (35) and the comparison of SJvJgx with SJvJsc2 (29). Comparisons between the three groups from Sichuan yielded only 15~24 unique differential metabolites. In summary, the metabolic differences of FBSJvJ were characterized primarily by inter-regional divergence, with phenolic acid glycosides emerging as shared biomarkers across origins. These observations imply that regional factors shape metabolite profiles [31]. Consistently, the three Sichuan-introduced groups showed relative metabolic homogeneity. Targeted follow-up studies (absolute quantitative validation and functional assays) are needed to confirm the biological relevance of these findings.

#### 3.5.1. Biomarker Screening Between the Guangxi Group and the Sichuan-Introduced Groups

**Figure 5:**
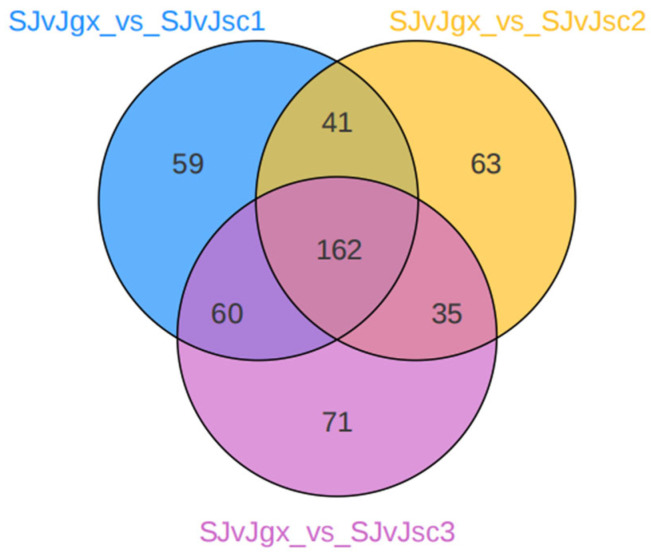
Venn diagram of shared and unique differential metabolites for SJvJgx compared to SJvJsc1–3.

We performed pairwise comparisons of SJvJgx with each Sichuan group to investigate metabolite differences between the Guangxi group (SJvJgx) and the three Sichuan-introduced groups (SJvJsc1–3). Using an OPLS-DA model and considering the thresholds of VIP ≥ 1, |log2FC| ≥ 1, and *p* ≤ 0.05, we detected 322, 301, and 328 differential metabolites when comparing SJvJgx with SJvJsc1, SJvJgx with SJvJsc2, and SJvJgx with SJvJsc3, respectively, yielding 492 differential metabolites in total (Figure 5). Among them, 162 metabolites were common to all three comparisons (Table S2), comprising 47 lipids, 27 nucleotides and their derivatives, 23 phenolic acids, 21 flavonoids, 20 amino acids and their derivatives, 5 organic acids, 4 alkaloids, 4 tannins, 1 lignan, 1 terpenoid, and 9 other metabolites. Among the 162 common metabolites, 15 were identified as TCM-KAIs and 15 were identified as PDRIs associated with the 11 diseases. In addition, 9 metabolites, including 2′-deoxyadenosine, punicic acid, cordycepin, digallic acid, apigenin-7,4′-dimethyl ether, salidroside, daidzin, medicarpin, and puerarin, were identified as both TCM-KAIs and PDRIs associated with the 11 diseases. The traditional quality evaluation of FBSJvJ mainly relies on the single index of rutin content, which lacks sufficient resolution in distinguishing origins and fine varieties [21]. The characteristic markers screened in this study exhibited high specificity and clear differential multiples. They offer potential values as supplementary indicators for origin traceability and variety identification [32] and provide a basis for a more comprehensive quality assessment of FBSJvJ. Due to the limited sampling scope in this study, the stability of these markers must be verified by multi-batch and multi-region samples.

**Figure 6:**
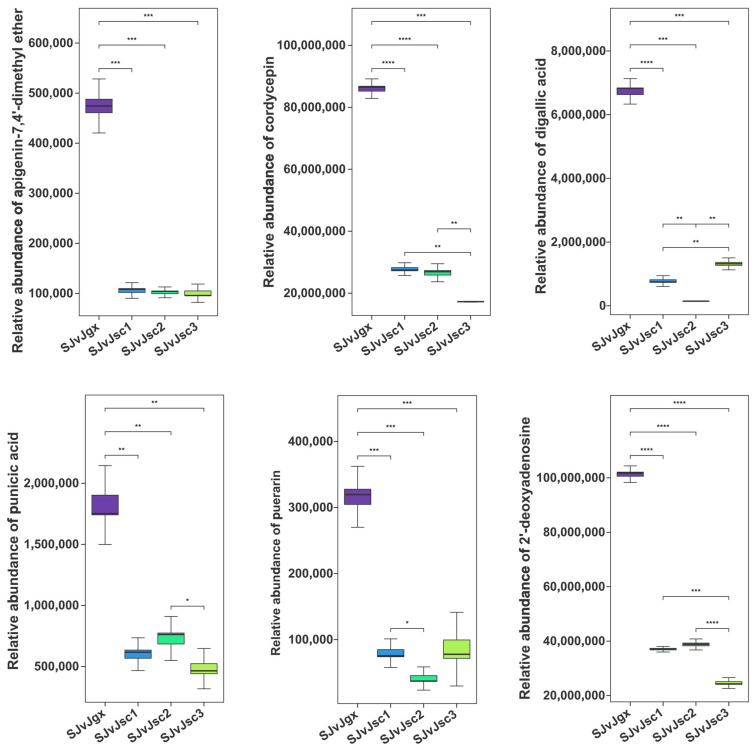
Boxplots of the 6 metabolites enriched in the SJvJgx group compared to the SJvJsc1–3 groups. Note: Group comparisons were conducted using the *t*-test: * *p* < 0.05, ** *p* < 0.01, *** *p* < 0.001, **** *p* < 0.0001.

Among the 162 shared differential metabolites, 49 were significantly more abundant in all three Sichuan-introduced groups (SJvJsc1–3) than in the Guangxi group (SJvJgx). These differential metabolites comprised 13 phenolic acids, 10 flavonoids, 7 amino acids and their derivatives, 7 nucleotides and their derivatives, 3 tannins, 2 alkaloids, 2 organic acids, and 5 other metabolites. Four out of the 49 metabolites were annotated as TCM-KAIs, and 4 were annotated as PDRIs associated with the 11 diseases. Three metabolites were identified as both TCM-KAIs and PDRIs. The 3 dual-annotated compounds included salidroside, daidzin, and medicarpin, whose relative abundances are presented in Figure 4B1–B3. Notably, the abundance patterns of daidzin and medicarpin across the four groups closely correspond to the morphological gradient in bud length and slenderness. Specifically, the abundance of daidzin and medicarpin was higher in more elongated/slender buds and lower in shorter, stouter ones. Conversely, 113 metabolites were consistently more abundant in Guangxi (SJvJgx) than in Sichuan. The 113 metabolites comprised 47 lipids, 20 nucleotides and their derivatives, 13 amino acids and their derivatives, 11 flavonoids, 10 phenolic acids, 3 organic acids, 2 alkaloids, 1 lignan, 1 tannin, 1 terpenoid, and 4 other metabolites. Among them, 11 metabolites were TCM-KAIs, 11 were PDRIs, and 12 satisfied both annotations. The 6 overlapping compounds comprised 2′-deoxyadenosine, cordycepin, punicic acid, digallic acid, apigenin-7,4′-dimethyl ether, and puerarin, whose relative abundances are presented in Figure 6. Notably, the Guangxi group contained more metabolites annotated as both TCM-KAIs and PDRIs.

#### 3.5.2. Differential Metabolites Among Sichuan-Introduced FBSJvJ Groups

**Figure 7:**
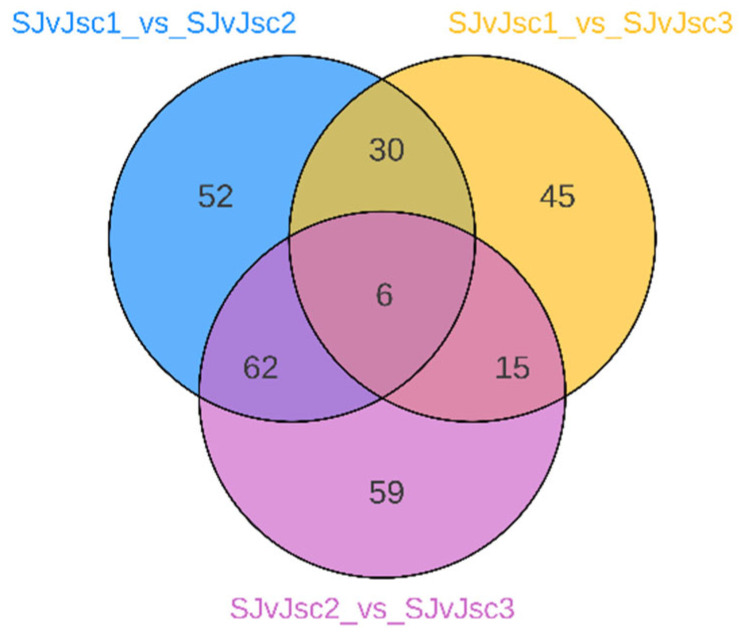
Venn diagram of differential metabolites among the SJvJsc1–3 groups.

Pairwise OPLS-DA comparisons were conducted using strict criteria (VIP ≥ 1, |log2FC| ≥ 1, and *p* ≤ 0.05) to resolve metabolic divergence among the three Sichuan-introduced FBSJvJ populations (SJvJsc1, SJvJsc2, and SJvJsc3). These pairwise comparisons yielded 150, 96, and 142 differential metabolites when comparing SJvJsc1 with SJvJsc2, SJvJsc1 with SJvJsc3, and SJvJsc2 with SJvJsc3, respectively. After integration and removal of duplicates across the three intra-Sichuan comparisons, 269 distinct differential metabolites were obtained. A Venn diagram (Figure 7) illustrates the distribution of shared and unique differentials and highlights the predominance of group-specific metabolites with relatively few shared features, consistent with only modest metabolic differentiation among the introduced Sichuan groups.

Each pairwise differential set was annotated against curated lists of TCM-KAIs and PDRIs, and metabolites annotated to both categories were further analyzed for expression trends. Comparison of SJvJsc1 with SJvJsc2 identified 19 differential metabolites matched to TCM-KAIs and 19 differential metabolites matched to PDRIs, with a subset annotated to both classes. Most of these dual-annotated metabolites exhibited downregulated trends in SJvJsc2 compared to SJvJsc1. Comparison of SJvJsc1 with SJvJsc3 identified 14 differential metabolites annotated as TCM-KAIs and 5 differential metabolites as PDRIs. Among them, there were three dual-annotated compounds, including irisolidone, betulinic acid, and ursolic acid, all of which were downregulated in SJvJsc1 compared to SJvJsc3. Comparison of SJvJsc2 with SJvJsc3 detected 18 differential metabolites as TCM-KAIs and nine differential metabolites as PDRIs, including six dual-annotated metabolites. Among them, scopolin, maackiain, and kaempferol were downregulated, while digallic acid, genistein-8-C-glucoside, and kaempferol-3,7-O-diglucoside were upregulated.

Collectively, these results suggest that although several bioactive candidates (TCM-KAIs and PDRIs) exist among Sichuan-introduced FBSJvJ groups, the expression changes remain largely group-specific and modest in terms of magnitude.

### 3.6. KEGG Metabolic Pathway Analysis of Differential Metabolites in FBSJvJ

Of the 49 metabolites significantly enriched in the Sichuan groups, 20 were KEGG-annotated and mapped to 46 pathways. Of the 113 metabolites significantly enriched in the Guangxi group, 19 were KEGG-annotated and mapped to 18 pathways. Moreover, among the 9 compounds annotated as both TCM-KAIs and PDRIs, four (2′-deoxyadenosine, salidroside, daidzin, and medicarpin) were KEGG-annotated and mapped to seven pathways, such as metabolic pathways (ko01100) and isoflavonoid biosynthesis (ko00943). The full list of the mapped pathways and KEGG IDs is provided in Table S3.

**Figure 8:**
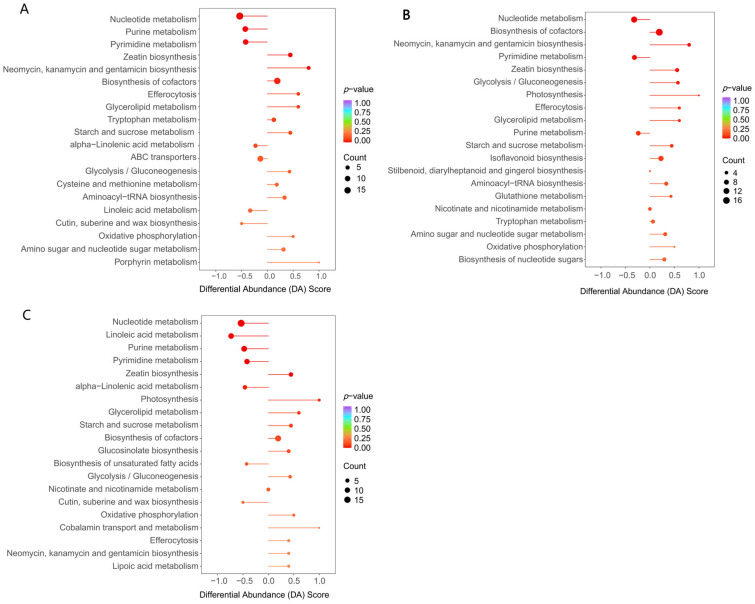
KEGG enrichment of different metabolites (DA score) in flower buds between SJvJgx and SJvJsc1 (**A**), between SJvJgx and SJvJsc2 (**B**), and between SJvJgx and SJvJsc3 (**C**).

DA > 0 indicated greater pathway activity in the Sichuan-introduced cultivars (SJvJsc1–3), and DA < 0 indicated greater activity in the Guangxi native population (SJvJgx). Pathway enrichment (*p* < 0.05) identified three pathways, including nucleotide metabolism (ko01232), pyrimidine metabolism (ko00240), and zeatin biosynthesis (ko00908), that were consistently significant across the three pairwise comparisons (Figure 8A–C). These results reflect a significant regional shift in metabolic allocation between the Guangxi group and the Sichuan-introduced groups. At the pathway level, SJvJgx showed significantly more active nucleotide and pyrimidine metabolism (DA < 0), whereas all Sichuan groups exhibited significantly greater zeatin biosynthesis (DA > 0). Detailed maps of these key pathways are presented in Figure S1 (ko00240), Figure S2 (ko01232), and Figure S3 (ko00908). These pathway maps visualize distinct regional patterns of metabolite distribution and reaction step enrichment across the three key modules.

Distinct Sichuan cultivars showed different adaptive strategies. SJvJsc1 and SJvJsc2 were enriched in the biosynthesis of cofactors and aminoglycosides (neomycin/kanamycin/gentamicin) (*p* < 0.05, DA > 0). SJvJsc2 was uniquely co-enriched in photosynthesis, glycolysis/gluconeogenesis, and glycerolipid metabolism (*p* < 0.05, DA > 0). By contrast, SJvJsc3 showed significant activation of photosynthesis (*p* < 0.05, DA > 0) but depletion in linoleic and α-linolenic acid metabolism (*p* < 0.05, DA < 0).

**Figure 9:**
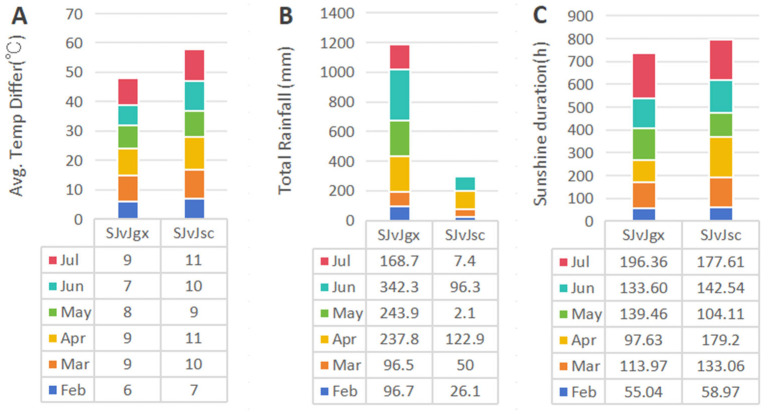
Column chart showing the average temperature difference (**A**), rainfall (**B**) and sunshine duration (**C**) in the two regions (February to July 2022).

Aggregated climate data from February to July (Figure 9) showed marked differences between the sampling sites of Sichuan and Guangxi. Compared to Guangxi locations, Sichuan locations were warmer, sunnier, and drier, with a cumulative mean monthly temperature of 58 °C vs. 48 °C, total sunshine duration of 795.5 h vs. 736.1 h, and cumulative rainfall of 304.8 mm vs. 1185.9 mm. These climatic differences were consistent with the observed metabolic and pathway divergence between the Guangxi group and the Sichuan FBSJvJ groups.

## 4. Discussions

Geographic origin strongly shapes the metabolome of medicinal plants. Using widely targeted metabolomics, this study compared FBSJvJ from Guangxi and three Sichuan-introduced cultivars to identify origin-specific markers. This study also explored functional metabolites (TCM-KAIs, PDRIs) and differential pathways correlated with local climate, supporting quality discrimination and rational exploitation of FBSJvJ. The sampling strategy followed the current planting distribution of SJvJ, with Guangxi as the core production area and Sichuan as a major introduced cultivation base. This representative sampling revealed clear metabolic divergence between native and introduced populations and provides baseline data to guide regional cultivation and industrial development.

Consistent with metabolome profiling and multivariate statistics in Section 3.1 and Section 3.4, PCA and HCA confirmed obvious metabolic divergence between the samples obtained from Guangxi and Sichuan, whereas three Sichuan cultivars exhibited high metabolomic similarities dominated by lipids, nucleotides, flavonoids, and phenolic acids. Using the obtained metabolite dataset, we screened TCM-KAIs and PDRIs via two public databases. Relevant bioactivity information was combined with the published literature collation and computational prediction. Though such an annotation preliminarily reflects incomplete database coverage, the screening facilitates preliminary bioactivity matching and provides a valuable reference for the quality control of FBSJvJ and resource exploitation, yet none of these annotated pharmacological activities were validated using in vitro or in vivo experiments, and their practical efficacy relies on further verification.

Phenolic acids (e.g., salvianolic acid) and flavonoids/isoflavonoids (e.g., daidzin, medicarpin) enriched in the Sichuan groups act as core defensive metabolites of plants against abiotic stress. Increased expression levels of these metabolites represent an antioxidant stress response of FBSJvJ to dry-hot and high-illumination environmental conditions in Sichuan [33]. In contrast, lipids (e.g., lysophospholipids) and nucleotide derivatives (e.g., cordycepin) enriched in the Guangxi group are closely associated with the structural stability of the cell membrane, nucleic acid synthesis, and energy metabolism [34]. This reflects the more vigorous vegetative growth characteristics of FBSJvJ under the high-moisture environment in Guangxi. Moreover, the enrichment of lysophospholipids may serve as a critical mechanism for adapting to high-humidity conditions and maintaining cell membrane permeability [35]. Comparative analysis between the Guangxi and three Sichuan groups revealed a consistent metabolic dichotomy. Although many individual metabolites are multifunctional or exhibit overlapping activities, the compositional differences suggest distinct functional tendencies. The Sichuan-enriched fraction is characterized chiefly by higher levels of phenolic acids and flavonoids. These classes of metabolites are frequently associated with antioxidant, anti-inflammatory, and defense-related activities [36,37]. The three compounds annotated as both TCM-KAIs and PDRIs in the Sichuan groups, namely salidroside, daidzin, and medicarpin, are consistent with this chemical-functional profile [38,39,40]. By contrast, the Guangxi group was found to be biased toward lipids and nucleotide-related metabolites, revealing a metabolic signature more closely linked to membrane composition, signalling, and energy/nucleotide metabolism. Dual-annotated Guangxi compounds, such as 2′-deoxyadenosine and cordycepin, highlight altered nucleotide metabolism and possess established antimicrobial, anti-inflammatory, and antitumor functions [41,42,43]. Similarly, punicic acid and other bioactive lipids underscore the metabolic-regulatory and anti-inflammatory function of lipids [44]. Polyphenols and methylated flavones detected in the Guangxi samples (e.g., digallic acid, apigenin derivatives) add antioxidant and antimicrobial capacities [45,46]. On the other hand, puerarin contributes documented cardioprotective and neuroprotective properties [47]. Collectively, the Guangxi profile suggests a greater capacity for modulating membrane-related processes and regulating systemic metabolism. Notably, more dual-annotated TCM-KAI/PDRI metabolites were detected in the Guangxi group, whereas non-lipid constituents were comparable across regions. Despite high metabolomic consistency across the three Sichuan cultivars, several dual-labeled compounds exhibited cultivar-specific accumulation patterns. Given the unvalidated actual content and pharmacological potency of these characteristic metabolites, future studies should conduct absolute quantification via LC–MS/MS and serial bioactivity assays, including antioxidant and anti-inflammatory tests. In addition, in vitro or in vivo experiments are needed to validate the biological functions of these metabolites.

KEGG enrichment analysis reflected distinct alterations in metabolic distribution between Guangxi and Sichuan-introduced groups. Pyrimidine and overall nucleotide metabolism were significantly enriched in Guangxi samples, while zeatin biosynthesis was markedly upregulated across all Sichuan cultivars. On the contrary, Sichuan cultivars accumulated abundant UDP-sugar and pyrimidine-derived precursors alongside elevated ATP/ADP pools (Figures S1–S3). These features support AtIPT-dependent trans-zeatin biosynthesis, and deceleration of pyrimidine degradation reduces unnecessary nucleotide consumption to reserve cytokinin precursors under local ambient conditions. Distinct pathway differentiation also existed among the three Sichuan cultivars. SJvJsc1 and SJvJsc2 were enriched in the biosynthesis of cofactors and aminoglycosides, which contributes to the formation of precursor supply for secondary metabolism and potential antimicrobial metabolites. SJvJsc2 uniquely upregulated photosynthesis, glycolysis/gluconeogenesis, and glycerolipid metabolism to enhance carbon fixation and membrane lipid remodeling for the production of highly consumed secondary metabolites. By contrast, SJvJsc3 activated photosynthesis but downregulated linoleic and α-linolenic acid metabolism, forming a unique resource allocation trade-off between energy production and polyunsaturated fatty acid biosynthesis [48]. Such metabolic differences between different groups from Sichuan may be attributable to variations in the field microenvironment, inherent genetic divergence of the introduced seedlings, and differences in the physicochemical properties of soil, rhizosphere microorganisms, epigenetic regulation, and anthropogenic influences reported in previous studies [49,50,51,52]. These cultivar-specific metabolic traits bring differentiated developmental prospects for targeted cultivation and deep processing of FBSJvJ resources.

Field meteorological data collected from February to July of the sampling year indicated apparent climatic disparities between the two planting regions. Compared with Guangxi, Sichuan possessed higher cumulative temperature and sunshine hours but much lower precipitation. Such climatic divergence is only one of several variables that may be correlated with the observed metabolic variation between different regions. Higher rainfall in Guangxi is correlated with accelerated nucleotide turnover and unsaturated fatty acid synthesis for maintaining cell membrane fluidity, whereas dry and high-sunlight surroundings in Sichuan may be associated with activation of the biosynthetic pathways of zeatin and isoflavonoid [53,54]. Enhanced flavonoid biosynthesis and UDP-mediated glycosylation can promote the accumulation of phenolic acids and flavonoids, most of which were annotated as both TCM-KAIs and PDRIs in our dataset. Synchronous elevation in the production of cofactor, ATP, and UDP can provide sufficient substrate for intensive secondary biosynthesis [53,54,55,56,57]. Nevertheless, the present study did not conduct continuous climate monitoring during several years; thus, these findings cannot establish a definitive causal relationship between climate and metabolic divergence or rule out the effects of other confounding factors, including genotype.

In this study, all metabolic and pathway inferences were based on relative metabolomic quantification and public database annotations; therefore, the results face inherent limitations. The sampling design, one native population from Guangxi versus three introduced lines from Sichuan, did not allow the full identification of genetic and environmental effects. Consequently, the observed inter-regional metabolic differences likely reflect the combined effects of genotype and local habitat rather than geographic climate alone. Samples and meteorological data were collected during a single growing season, making the assessment of temporal stability impossible. Future studies should conduct the absolute quantification of key metabolites using targeted LC-MS/MS with authentic standards, validate metabolite identities and pathway assignments, quantify the expression of key biosynthetic genes using qPCR and/or transcriptomics, and perform reciprocal-transplant/common-garden experiments combined with multi-site, multi-year sampling to disentangle genetic and environmental contributions. In the absence of such validation studies, causal links between climate and metabolic variation should be interpreted cautiously.

In summary, the environmental conditions of the growing regions are closely correlated with the primary and secondary metabolic remodeling of FBSJvJ. The characterized regional metabolic features and high levels of flavonoid and phenolic acid components identified in this work lay practical foundations for standardized planting, contribute to precise quality grading, and facilitate the development of FBSJvJ-derived functional foods.

## 5. Conclusions

In this study, UPLC-MS/MS-based widely targeted metabolomics identified 1550 metabolites across 12 classes from one-season harvested FBSJvJ. In total, 152 TCM-KAIs and 204 disease-related candidates were screened via public databases. Clear metabolic divergence existed between the two cultivation regions. Specifically, Guangxi samples accumulated higher levels of lipids and nucleotides, whereas three Sichuan-introduced cultivars exhibited higher levels of flavonoids and phenolic acids, corresponding to distinct regulation of pyrimidine metabolism and zeatin biosynthesis. While the three Sichuan cultivars shared similar overall metabolomic profiles, cultivar-specific variations in metabolism and pathways were detected. These findings support discrimination in geographical origin, contribute to the routine quality assessment of this edible and medicinal plant, and provide a reference for germplasm selection, targeted cultivation, and development of functional foods. Further multi-site and multi-year sampling combined with multi-omics analysis is required to clarify the effects of genotype and environment on metabolic variation.

## Figures and Tables

**Table 1:** The TCM-KAIs of FBSJvJ exhibiting OB ≥ 5% and DL ≥ 0.14.

Classification	Substance Name
Phenolic acids	6-O-Galloyl-β-D-glucose *; Isotachioside; Cryptochlorogenic acid *; Arbutin; Androsin, Neochlorogenic acid *; Digallic acid; Salidroside; Chlorogenic acid *; Bis(2-ethylhexyl)phthalate *; Isomartynoside
Nucleotides and their derivatives	2′-Deoxyadenosine; Guanosine; Cordycepin; Adenosine; Uridine 5′-diphospho-D-glucose; Uridine 5′-monophosphate; Inosine
Flavonoids	Ononin; Hispidulin; Diosmetin; Isorhamnetin; 3′-Methoxy-3,4′,5,7-Tetrahydroxyflavone; Robinin; Afrormosin; Naringenin; Pectolinarigenin; Calycosin-7-O-glucoside; Biochanin A; 3′-Methoxydaidzein; Taxifolin; Sophoraflavanone G; 5,7,4′-Trihydroxy-3′-methoxyisoflavone; 3′-O-Methylorobol; Homoplantaginin; Maackiain; Tectorigenin; Wogonin; Luteolin; Apigenin; 4′,5,7-Trihydroxyflavone; Quercetin; Cirsimaritin *; Genistein; Morin; Isoluteolin; Vestitol; Glycitein; 7,4′-Di-O-methyldaidzein; pseudobaptigenin; Astrapterocarpan; Irisolidone; Acacetin; Chrysoeriol-7-O-glucoside; Glycitin; 8-O-methylretusin; Genistin; 6″-O-Malonylglycitin; Kaempferol-7-O-rhamnoside; Isoliquiritin; Pterocarpine; Glycyroside; Eriodictyol-7-O-glucoside; Apigenin-7,4′-dimethyl ether; Formononetin; Kaempferol; Eriodictyol; Genistein-8-C-glucoside; Sexangularetin; 6-Hydroxyluteolin; Catechin; Daidzin; 3′,4′,7-Trihydroxyflavone; Isoquercitrin *; Claussequinone; Sissotrin; Okanin; Glabranine; 3,5,6,7,8,3′,4′-Heptamethoxyflavone; Isoorientin; Epicatechin gallate *; Dihydrokaempferol; Garbanzol; Limocitrin; Medicarpin; Liquiritigenin; 7,4′-Dihydroxyflavone; Prunin; Quercetin-3-O-(2″-O-galactosyl)glucoside; Kaempferol-3,7-O-diglucoside; 5,2′-Dihydroxy-7,8-dimethoxyflavone; Choerospondin; Isobavachin; Liquiritin; Isorhamnetin-3,7-O-diglucoside; 6″-O-Malonyldaidzin; Trifolirhizin; Sophoricoside; 6″-O-Acetylgenistin; 4,4′-Dihydroxy-2-methoxychalcone; Echinatin; Daidzein; Vitexin; Cosmosiin; Nepitrin; 7-O-Methyleriodictyol; Astilbin; 7,3′,4′-Trihydroxyflavone; Kanzonol H; 6″-O-Acetylglycitin; Luteolin-7,3′-di-O-glucoside *; Puerarin; Salvigenin; Eupatilin; Kaempferide; Linarin; Vitexin-2″-O-rhamnoside; Rutin *
Quinones	Embelin; Aloe emodin
Others	Capillarisin; Riboflavin; Icariside E5; Pentadecanoic acid, 14-methyl-,methyl ester; 5-O-Methylvisammioside
Lignans and coumarins	Scopolin; Stevenin; Esculin; Dehydrodiconiferyl alcohol; Syringaresinol-4′-O-glucoside; Acanthoside B; Olivil-4′-O-glucoside
Tannins	Procyanidin B1
Terpenes	Soyasapogenol B; 23-Hydroxybetulinic acid; Betulinic acid; Soyasaponin I; Corosolic acid methyl ester; Ursolic acid; Soyasapogenol E; Dehydrosoyasaponin I *; Rubiatriol
Lipids	Ricinoleic acid; 9-Hydroxy-10,12,15-octadecatrienoic acid; Punicic acid; 1-Eicosanol; γ-Linolenic Acid *; α-Linolenic Acid *; Hexadecanedioic acid; Vaccenic acid *; Petroselinic acid *; Methyl linolenate; Gingerglycolipid B; 1-Linoleoylglycerol *; Lignoceric acid; Gingerglycolipid A; Eicosenoic acid

Note: * means isomers.

## Data Availability

Data are provided within the manuscript or Supplementary Materials files. The experimental data and the simulation results supporting the findings of this study were obtained from the original research. Qualitative analysis of substances was based on the criteria of fragmentation pattern, retention time, Metware database (MWDB) V2.0, and public database established by *m*/*z* and Metware Biotechnology Co., Ltd. (Wuhan, China). The statistical analysis and figure preparation were conducted using the appropriate software: R program Version 2.8.0 for Complex Heatmap, R program Version 0.84 for Corrplot, and R program Version 0.84 for MetaboAnalystR. All data supporting this study related to the TCMSP and CancerHSP databases can be accessed via https://www.tcmsp-e.com/#/home (accessed on 2 July 2026). All data supporting this study related to the KEGG database can be accessed via http://www.kegg.jp/kegg/compound/ (accessed on 2 July 2026) and http://www.kegg.jp/kegg/pathway.html (accessed on 2 July 2026). Climatic data (temperature, rainfall, and sunshine duration) for the four production regions from February to May 2022 were obtained from publicly available sources. Temperature and rainfall data were obtained from https://www.tianqi24.com (accessed on 2 July 2026), and sunshine duration data were obtained from https://www.ceicdata.com/zh-hans/china/sunshine-hours (accessed on 2 July 2026).

## Supplementary Materials

The following supporting information can be downloaded at: https://www.mdpi.com/article/10.3390/metabo16070475/s1, Table S1: Summary of FBSJvJ PDRIs against 11 human diseases as reported in the CancerHSP and TCMSP database; Table S2: 162 differential metabolites identified through pairwise comparison (SJvJgx vs. SJvJsc3, SJvJgx vs. SJvJsc1, SJvJgx vs. SJvJsc2); Table S3: KEGG annotation and pathway mapping for 39 KEGG-annotated metabolites of 162 shared differential metabolites in all comparisons between the FBSJvJ groups (20 upregulated and 19 downregulated); Figure S1: Differential metabolites KEGG pathway map of isoflavonoid biosynthesis (ko00943) in SJsd vs. SJah; Figure S2: KEGG pathway graph of differential metabolites from pyrimidine metabolism when comparing SJvJgx with SJvJsc1(ko00240); Figure S3: KEGG pathway graph of differential metabolites from nucleotide metabolism (ko01232) when comparing SJvJgx with SJvJsc1.
