# Supplementary material for: Geographical Origin Drives Metabolic Divergence in Styphnolobium japonicum cv. Jinhuai: A Widely Targeted Metabolomic Study of Flower Buds from Sichuan and Guangxi, China
Source: Metabolites. 2026 Jul 7;16(7):475. doi: 10.3390/metabo16070475 (PMC13414312; doi:10.3390/metabo16070475)
Supplement: Supplementary file 1 [file metabolites-16-00475-s001.zip › metabolites-4298900-supplementary.pdf]

Table S1. Summary of FBSJvJ PDRIs against 11 human diseases as reported in the CancerHSP and TCMSP database

Diseases		Components
6 CDs	Cancer/tumor	Sinapinaldehyde; Vanillic acid; Caffeic acid; Gallic acid; Salidroside; Vanillin; 4-Hydroxy-3-Methoxybenzaldehyde; p-Coumaric acid; 3,4-Dihydroxybenzoic acid (Protocatechuic acid)*; Chlorogenic acid (3-O-Caffeoylquinic acid)*; Hydroquinone; Methyl caffeate; Cordycepin (3'-Deoxyadenosine); Phloretin; Liquiritigenin; Isobavachin; Liquiritigenin-4'-O-Glucoside (Liquiritin); Naringenin-7-O-Neohesperidoside(Naringin); Aromadendrin (Dihydrokaempferol); Taxifolin-3-O-rhamnoside (Astilbin); Hispidulin (5,7,4'-Trihydroxy-6-methoxyflavone); Diosmetin (5,7,3'-Trihydroxy-4'-methoxyflavone); Wogonin (5,7-Dihydroxy-8-Methoxyflavone); Apigenin; 4',5,7-Trihydroxyflavone; Cirsimaritin (4',5-dihydroxy-6,7-dimethoxyflavone)*; Acacetin; Apigenin-8-C-Glucoside (Vitexin); Apigenin-7-O-glucoside(Cosmosiin); Quercetin-3-O-rutinoside (Rutin)*; Quercetin; Morin; Kaempferol (3,5,7,4'-Tetrahydroxyflavone); Quercetin-3-O-galactoside (Hyperin); Quercetin-3-O-rhamnoside(Quercitrin); Catechin; 7-O-Methyleriodictyol; Claussequinone; Biochanin A; Tectorigenin; Genistein; 7,4'-Di-O-methyldaidzein; Pterocarpine; Formononetin (7-Hydroxy-4'-methoxyisoflavone); Medicarpin; Daidzein; Embelin; 2,6-Dimethoxy-1,4-benzoquinone; Coumarin; Procyanidin B1; Procyanidin B2*; 3,23-Dihydroxylup-20(29)-en-28-oic acid (23-Hydroxybetulinic acid); 3-Hydroxylup-20(29)-en-28-oic acid (Betulinic acid); 3-Hydroxyurs-12-en-28-oic acid (Ursolic acid); Azukisaponin VI; cis-Citral; Cis-Aconitic acid; Fumaric acid
	Diabetes	Dihydroferulic Acid; Cinnamic acid; Neochlorogenic acid (5-O-Caffeoylquinic acid)*; Digallic Acid; Gallic acid; Hydrocinnamic acid; 3,4-Dihydroxybenzoic acid (Protocatechuic acid)*; Hydroquinone; Phloretin-2'-O-glucoside (Phlorizin); 4,4'-Dihydroxy-2-methoxychalcone; Echinatin; Sophoraflavanone G; Taxifolin(Dihydroquercetin); Hispidulin (5,7,4'-Trihydroxy-6-methoxyflavone); Diosmetin (5,7,3'-Trihydroxy-4'-methoxyflavone); Pectolinarigenin; Wogonin (5,7-Dihydroxy-8-Methoxyflavone); Luteolin (5,7,3',4'-Tetrahydroxyflavone); Cirsimaritin (4',5-dihydroxy-6,7-dimethoxyflavone)*; Luteolin-7-o-glucuronide; Acacetin; 4',5,7-Trihydroxy-3',6-dimethoxyflavone (Jaceosidin); Luteolin-6-C-glucoside (Isoorientin); 5,2'-Dihydroxy-7,8-dimethoxyflavone; 5-Hydroxy-4',6,7-trimethoxyflavone (Salvigenin); Eupatilin (5,7-Dihydroxy-3',4',6-Trimethoxyflavone); Isorhamnetin; 3'-Methoxy-3,4',5,7-Tetrahydroxyflavone; Quercetin-3-O-rutinoside (Rutin)*; Quercetin; Morin; Rhamnetin-3-O-rhamnoside; Kaempferol (3,5,7,4'-Tetrahydroxyflavone); Sexangularetin; 3,5,6,7,8,3',4'-Heptamethoxyflavone; Limocitrin (5,7,4'-trihydroxy-8,3'-dimethoxyflavone); Kaempferol-3-O-glucuronide; Kaempferide (3,5,7-Trihydroxy-4'-methoxyflavone); Vestitol; Claussequinone; Formononetin-7-O-glucoside (Ononin); Afrormosin (6,4'-Dimethoxy-7-Hydroxyisoflavone); Biochanin A; 3'-Methoxydaidzein; 5,7,4'-Trihydroxy-3'-methoxyisoflavone; 3'-O-Methylorobol; Tectorigenin; Genistein; Glycitein; pseudobaptigenin; Irisolidone; 8-O-methylretusin; Formononetin (7-Hydroxy-4'-methoxyisoflavone); Genistein-8-C-glucoside; Daidzein-7-O-glucoside(Daidzin); Medicarpin; Daidzein; 6''-O-Acetylglycitin; Puerarin (7,4'-Dihydroxy-8-C-glucosylisoflavone); Aloe emodin; Dehydrodiconiferyl alcohol; Scopoletin-7-O-glucoside (Scopolin); stevenin; Esculetin (6,7-Dihydroxycoumarin); Capillarisin; beta-D-Galactose; Indole-3-acetic acid (IAA); DL-Glyceric Acid; Succinic acid; Phenylpyruvic acid; L-Malic acid; Citric Acid; cis-Citral; Fumaric acid; L-Tartaric acid; DL-3-Phenyllactic acid*; Creatine; α-Linolenic Acid*
	Hypertension	L-Citrulline; L-Homocitrulline; L-Leucine; L-Tyrosine; Dimethyl phthalate; Isovanillin; Dihydroferulic Acid; 3-Hydroxy-4-methoxybenzoic acid; Isovanillic Acid; Cinnamic acid; Cryptochlorogenic acid (4-O-Caffeoylquinic acid)*; Androsin; Salicylic acid; Sinapinaldehyde; Protocatechuic Acid Methyl Ester; Vanillic acid; Methyl gallate; Caffeic acid; Diisobutyl phthalate; Digallic Acid; Gallic acid; Hydrocinnamic acid; Salidroside; Vanillin; 4-Hydroxy-3-Methoxybenzaldehyde; 3,4-Dihydroxybenzoic acid (Protocatechuic acid)*; Syringaldehyde; 4-Hydroxy-3,5-Dimethoxybenzaldehyde; Chlorogenic acid (3-O-Caffeoylquinic acid)*; 2-Hydroxycinnamic acid*; Sinapic acid; 3,4-Dimethoxycinnamic acid; 2,6-Dimethoxybenzoic acid; 2,5-Dihydroxybenzoic acid; Gentisic

Diseases		Components
		Acid*; Ferulic acid; Ethylparaben; Tyrosol; 4-Hydroxyphenylethanol; Methyl 4-hydroxybenzoate; Syringic acid; Methyl caffeate; 1-beta-D-Arabinofuranosyluracil; 2'-Deoxyadenosine; Cordycepin (3'-Deoxyadenosine); 4,4'-Dihydroxy-2-methoxychalcone; Echinatin; Naringenin (5,7,4'-Trihydroxyflavanone); Sophoraflavanone G; Eriodictyol (5,7,3',4'-Tetrahydroxyflavanone); Glabranine; Liquiritigenin; Naringenin-7-O-glucoside (Prunin); Choerospondin; Isobavachin; Liquiritigenin-4'-O-Glucoside (Liquiritin); Naringenin-7-O-Neohesperidoside(Naringin); Taxifolin(Dihydroquercetin); Aromadendrin (Dihydrokaempferol); Taxifolin-3-O-rhamnoside (Astilbin); Hispidulin (5,7,4'-Trihydroxy-6-methoxyflavone); Diosmetin (5,7,3'-Trihydroxy-4'-methoxyflavone); Pectolinarigenin; Wogonin (5,7-Dihydroxy-8-Methoxyflavone); Luteolin (5,7,3',4'-Tetrahydroxyflavone); Cirsimaritin (4',5-dihydroxy-6,7-dimethoxyflavone)*; Acacetin; Apigenin-7,4'-dimethyl ether; 6-Hydroxyluteolin; 4',5,7-Trihydroxy-3',6-dimethoxyflavone (Jaceosidin); 5,2'-Dihydroxy-7,8-dimethoxyflavone; Apigenin-8-C-Glucoside (Vitexin); Apigenin-7-O-glucoside(Cosmosiin); 5-Hydroxy-4',6,7-trimethoxyflavone (Salvigenin); Eupatilin (5,7-Dihydroxy-3',4',6-Trimethoxyflavone); Isorhamnetin; 3'-Methoxy-3,4',5,7-Tetrahydroxyflavone; Quercetin; Morin; Kaempferol (3,5,7,4'-Tetrahydroxyflavone); Sexangularetin; Kaempferol-7-O-glucoside; 3,5,6,7,8,3',4'-Heptamethoxyflavone; Garbanzol; Limocitrin (5,7,4'-trihydroxy-8,3'-dimethoxyflavone); Kaempferol-3-O-arabinoside (Juglanin); Kaempferide (3,5,7-Trihydroxy-4'-methoxyflavone); Catechin; 7-O-Methyleriodictyol; Vestitol; Astrapterocarpan; Claussequinone; Formononetin-7-O-glucoside (Ononin); Afrormosin (6,4'-Dimethoxy-7-Hydroxyisoflavone); Calycosin-7-O-glucoside; Biochanin A; 3'-Methoxydaidzein; 5,7,4'-Trihydroxy-3'-methoxyisoflavone; 3'-O-Methylorobol; Maackiain; Tectorigenin; Genistein; Glycitein; pseudobaptigenin; Irisolidone; Glycitin; 8-O-methylretusin; Genistein-7-O-Glucoside (Genistin); Pterocarpine; Formononetin (7-Hydroxy-4'-methoxyisoflavone); Daidzein-7-O-glucoside(Daidzin); Medicarpin; Trifolirhizin (Maackiain-3-O-glucoside); Sophoricoside; Daidzein; Puerarin (7,4'-Dihydroxy-8-C-glucosylisoflavone); Aloe emodin; Embelin; Epipinoresinol; Dehydrodiconiferyl alcohol; Scopoletin-7-O-glucoside (Scopolin); stevenin; Esculin (6,7-Dihydroxycoumarin-6-O-glucoside); 3,4-Dihydrocoumarin; Coumarin; Eucommiol; Capillarisin; 5,7-Dihydroxychromone; beta-D-Galactose; Stachyose; 1,5-Anhydro-D-glucitol; Nicotinamide; Pyridoxine; Riboflavin (Vitamin B2); Procyanidin B1; Procyanidin B2*; Candicine; L-Tyramine; Tryptamine; Abrine; Indole-3-acetic acid (IAA); 4-Guanidinobutyric acid; Shikimic acid; Phenylpyruvic acid; Quinic Acid; Citric Acid; Abscisic acid; Cis-Aconitic acid; Fumaric acid; L-Tartaric acid; DL-3-Phenyllactic acid*; Ricinoleic acid; 9-Hydroxy-10,12,15-octadecatrienoic acid; Punicic acid (9Z,11E,13Z-octadecatrienoic acid); γ-Linolenic Acid*; α-Linolenic Acid*; Dodecanoic acid (Lauric acid); Methyl linolenate; Pentadecanoic Acid
	Cardiovascular Disease	L-Leucine; Isovanillin; Dihydroferulic Acid; 3-Hydroxy-4-methoxybenzoic acid; Isovanillic Acid; Cinnamic acid; Salicylic acid; Sinapinaldehyde; Protocatechuic Acid Methyl Ester; Vanillic acid; Methyl gallate; Caffeic acid; Gallic acid; Salidroside; 3,4-Dihydroxybenzoic acid (Protocatechuic acid)*; Syringaldehyde; 4-Hydroxy-3,5-Dimethoxybenzaldehyde; 2-Hydroxycinnamic acid*; Sinapic acid; 3,4-Dimethoxycinnamic acid; 2,6-Dimethoxybenzoic acid; 2,5-Dihydroxybenzoic acid; Gentisic Acid*; Ferulic acid; Ethylparaben; Tyrosol; 4-Hydroxyphenylethanol; Methyl 4-hydroxybenzoate; Methyl caffeate; 2'-Deoxyadenosine; Cordycepin (3'-Deoxyadenosine); Isoliquiritin; 4,4'-Dihydroxy-2-methoxychalcone; Echinatin; Naringenin (5,7,4'-Trihydroxyflavanone); Sophoraflavanone G; Eriodictyol (5,7,3',4'-Tetrahydroxyflavanone); Glabranine; Liquiritigenin; Isobavachin; Liquiritigenin-4'-O-Glucoside (Liquiritin); Naringenin-7-O-Neohesperidoside(Naringin); Taxifolin(Dihydroquercetin); Aromadendrin (Dihydrokaempferol); Taxifolin-3-O-rhamnoside (Astilbin); Hispidulin (5,7,4'-Trihydroxy-6-methoxyflavone); Diosmetin (5,7,3'-Trihydroxy-4'-methoxyflavone); Pectolinarigenin; Wogonin (5,7-Dihydroxy-8-Methoxyflavone); Luteolin (5,7,3',4'-Tetrahydroxyflavone); Cirsimaritin (4',5-dihydroxy-6,7-dimethoxyflavone)*; Luteolin-7-o-glucuronide; Acacetin; Apigenin-7,4'-

Diseases		Components
		dimethyl ether; 6-Hydroxyluteolin; 4',5,7-Trihydroxy-3',6-dimethoxyflavone (Jaceosidin); 5,2'-Dihydroxy-7,8-dimethoxyflavone; Nepetin-7-O-glucoside(Nepitrin); 5-Hydroxy-4',6,7-trimethoxyflavone (Salvigenin); Eupatilin (5,7-Dihydroxy-3',4',6-Trimethoxyflavone); Isorhamnetin; 3'-Methoxy-3,4',5,7-Tetrahydroxyflavone; Quercetin; Morin; Kaempferol (3,5,7,4'-Tetrahydroxyflavone); Sexangularetin; 3,5,6,7,8,3',4'-Heptamethoxyflavone; Garbanzol; Limocitrin (5,7,4'-trihydroxy-8,3'-dimethoxyflavone); Kaempferol-3-O-glucuronide; Kaempferol-3-O-arabinoside (Juglanin); Quercetin-3-O-rhamnoside(Quercitrin); Kaempferide (3,5,7-Trihydroxy-4'-methoxyflavone); Catechin; 7-O-Methyleriodictyol; Vestitol; Astrapterocarpan; Claussequinone; Formononetin-7-O-glucoside (Ononin); Afrormosin (6,4'-Dimethoxy-7-Hydroxyisoflavone); Biochanin A; 3'-Methoxydaidzein; 5,7,4'-Trihydroxy-3'-methoxyisoflavone; 3'-O-Methylorobol; Maackiain; Tectorigenin; Genistein; Glycitein; pseudobaptigenin; Irisolidone; 8-O-methylretusin; Pterocarpine; Formononetin (7-Hydroxy-4'-methoxyisoflavone); Daidzein-7-O-glucoside(Daidzin); Medicarpin; Daidzein; Puerarin (7,4'-Dihydroxy-8-C-glucosylisoflavone); Aloe emodin; Epipinoresinol; Dehydrodiconiferyl alcohol; stevenin; 3,4-Dihydrocoumarin; Capillarisin; 5,7-Dihydroxychromone; 5-O-Methylvisammioside; beta-D-Galactose; Stachyose; 1,5-Anhydro-D-glucitol; Nicotinamide; Pyridoxine; Procyanidin B1; Candicine; L-Tyramine; Tryptamine; Abrine; Indole-3-acetic acid (IAA); 4-Guanidinobutyric acid; DL-Glyceric Acid; Phenylpyruvic acid; Citric Acid; Fumaric acid; L-Tartaric acid; Malonic acid; DL-3-Phenyllactic acid*; 1-Linoleoylglycerol*; Ricinoleic acid; 9-Hydroxy-10,12,15-octadecatrienoic acid; Punicic acid (9Z,11E,13Z-octadecatrienoic acid); γ-Linolenic Acid*; α-Linolenic Acid*; Palmitoleic Acid; Dodecanoic acid (Lauric acid); 11-Octadecanoic acid(Vaccenic acid)*; Petroselinic acid*; Methyl linolenate; Pentadecanoic Acid
	Atherosclerosis	Dihydroferulic Acid; Cinnamic acid; Salidroside; Isoliquiritin; 4,4'-Dihydroxy-2-methoxychalcone; Echinatin; Naringenin (5,7,4'-Trihydroxyflavanone); Sophoraflavanone G; Glabranine; Liquiritigenin; Isobavachin; Naringenin-7-O-Neohesperidoside(Naringin); Hispidulin (5,7,4'-Trihydroxy-6-methoxyflavone); Pectolinarigenin; Wogonin (5,7-Dihydroxy-8-Methoxyflavone); 4',5,7-Trihydroxy-3',6-dimethoxyflavone (Jaceosidin); 5-Hydroxy-4',6,7-trimethoxyflavone (Salvigenin); Eupatilin (5,7-Dihydroxy-3',4',6-Trimethoxyflavone); Isorhamnetin; 3'-Methoxy-3,4',5,7-Tetrahydroxyflavone; Quercetin; Morin; Kaempferol (3,5,7,4'-Tetrahydroxyflavone); Kaempferol-7-O-glucoside; 3,5,6,7,8,3',4'-Heptamethoxyflavone; Limocitrin (5,7,4'-trihydroxy-8,3'-dimethoxyflavone); Kaempferide (3,5,7-Trihydroxy-4'-methoxyflavone); Catechin; Vestitol; Astrapterocarpan; Formononetin-7-O-glucoside (Ononin); Afrormosin (6,4'-Dimethoxy-7-Hydroxyisoflavone); Calycosin-7-O-glucoside; Biochanin A; 3'-Methoxydaidzein; 5,7,4'-Trihydroxy-3'-methoxyisoflavone; 3'-O-Methylorobol; Tectorigenin; Genistein; Glycitein; pseudobaptigenin; Irisolidone; Glycitin; 8-O-methylretusin; Genistein-7-O-Glucoside (Genistin); Formononetin (7-Hydroxy-4'-methoxyisoflavone); Daidzein-7-O-glucoside(Daidzin); Medicarpin; Daidzein; 6''-O-Acetylglycitin; Puerarin (7,4'-Dihydroxy-8-C-glucosylisoflavone); Embelin; Dehydrodiconiferyl alcohol; Coumarin; 5,7-Dihydroxychromone; Phenylpyruvic acid; DL-3-Phenyllactic acid*
	Thrombotic Diseases	Dihydroferulic Acid; Cinnamic acid; Diisobutyl phthalate; 4,4'-Dihydroxy-2-methoxychalcone; Echinatin; Sophoraflavanone G; Glabranine; Isobavachin; Liquiritigenin-4'-O-Glucoside (Liquiritin); Hispidulin (5,7,4'-Trihydroxy-6-methoxyflavone); Pectolinarigenin; Wogonin (5,7-Dihydroxy-8-Methoxyflavone); Luteolin-7-o-glucuronide; 4',5,7-Trihydroxy-3',6-dimethoxyflavone (Jaceosidin); Nepetin-7-O-glucoside(Nepitrin); 5-Hydroxy-4',6,7-trimethoxyflavone (Salvigenin); Eupatilin (5,7-Dihydroxy-3',4',6-Trimethoxyflavone); Quercetin; Kaempferol (3,5,7,4'-Tetrahydroxyflavone); Kaempferol-7-O-glucoside; 3,5,6,7,8,3',4'-Heptamethoxyflavone; Limocitrin (5,7,4'-trihydroxy-8,3'-dimethoxyflavone); Kaempferol-3-O-glucuronide; Kaempferol-3-O-arabinoside (Juglanin); Quercetin-3-O-rhamnoside(Quercitrin); Kaempferide (3,5,7-Trihydroxy-4'-methoxyflavone); Vestitol; Astrapterocarpan; Formononetin-7-O-glucoside (Ononin); Afrormosin (6,4'-Dimethoxy-7-Hydroxyisoflavone); Calycosin-7-O-

Diseases		Components
		glucoside; Biochanin A; 3'-Methoxydaidzein; 5,7,4'-Trihydroxy-3'-methoxyisoflavone; 3'-O-Methylorobol; Tectorigenin; Genistein; Glycitein; pseudobaptigenin; Irisolidone; Glycitin; 8-O-methylretusin; Genistein-7-O-Glucoside (Genistin); Formononetin (7-Hydroxy-4'-methoxyisoflavone); Daidzein-7-O-glucoside(Daidzin); Medicarpin; Daidzein; 6''-O-Acetylglycitin; Embelin; Epipinoresinol; Dehydrodiconiferyl alcohol; Coumarin; Capillarisin; 5,7-Dihydroxychromone; 5-O-Methylvisammioside; 4-Guanidinobutyric acid; Phenylpyruvic acid; Citric Acid; Fumaric acid; DL-3-Phenyllactic acid*
5 ChP–related diseases	Osteoporosis	Salicylic acid; Salidroside; Isoliquiritin; 4,4'-Dihydroxy-2-methoxychalcone; Echinatin; Naringenin (5,7,4'-Trihydroxyflavanone); Sophoraflavanone G; Glabranine; Liquiritigenin; Isobavachin; Naringenin-7-O-Neohesperidoside(Naringin); Wogonin (5,7-Dihydroxy-8-Methoxyflavone); Luteolin (5,7,3',4'-Tetrahydroxyflavone); 4',5,7-Trihydroxy-3',6-dimethoxyflavone (Jaceosidin); Eupatilin (5,7-Dihydroxy-3',4',6-Trimethoxyflavone); Quercetin-3-O-rutinoside (Rutin)*; Quercetin; 3,5,6,7,8,3',4'-Heptamethoxyflavone; Limocitrin (5,7,4'-trihydroxy-8,3'-dimethoxyflavone); Kaempferide (3,5,7-Trihydroxy-4'-methoxyflavone); Catechin; Vestitol; Astrapterocarpan; Formononetin-7-O-glucoside (Ononin); Afrormosin (6,4'-Dimethoxy-7-Hydroxyisoflavone); Biochanin A; 3'-Methoxydaidzein; 5,7,4'-Trihydroxy-3'-methoxyisoflavone; 3'-O-Methylorobol; Tectorigenin; Genistein; Glycitein; pseudobaptigenin; Irisolidone; 8-O-methylretusin; Formononetin (7-Hydroxy-4'-methoxyisoflavone); Daidzein-7-O-glucoside(Daidzin); Medicarpin; Daidzein; Puerarin (7,4'-Dihydroxy-8-C-glucosylisoflavone); Dehydrodiconiferyl alcohol; beta-D-Galactose; Indole; DL-Glyceric Acid; Succinic acid; L-Malic acid; Citric Acid; Fumaric acid; Malonic acid; Dodecanoic acid (Lauric acid)
	Hemorrhage	Salicylic acid
	Liver Ischemic Injury	Salicylic acid; Liquiritigenin-4'-O-Glucoside (Liquiritin); Quercetin-3-O-rutinoside (Rutin)*; Quercetin; Puerarin (7,4'-Dihydroxy-8-C-glucosylisoflavone)
	Inflammation	L-Citrulline; L-Homocitrulline; L-Leucine; L-Tyrosine; Dimethyl phthalate; Isovanillin; 4-Hydroxybenzoic acid; Dihydroferulic Acid; 3-Hydroxy-4-methoxybenzoic acid; Isovanillic Acid; Cinnamic acid; Cryptochlorogenic acid (4-O-Caffeoylquinic acid)*; Androsin; Salicylic acid; Sinapinaldehyde; Protocatechuic Acid Methyl Ester; Vanillic acid; Protocatechualdehyde; Methyl gallate; Caffeic acid; Diisobutyl phthalate; Digallic Acid; Gallic acid; Hydrocinnamic acid; Salidroside; Vanillin; 4-Hydroxy-3-Methoxybenzaldehyde; 3,4-Dihydroxybenzoic acid (Protocatechuic acid)*; Syringaldehyde; 4-Hydroxy-3,5-Dimethoxybenzaldehyde; Chlorogenic acid (3-O-Caffeoylquinic acid)*; 2-Hydroxycinnamic acid*; Sinapic acid; 3,4-Dimethoxycinnamic acid; 2,6-Dimethoxybenzoic acid; 2,5-Dihydroxybenzoic acid; Gentisic Acid*; Ferulic acid; Ethylparaben; Methyl 4-hydroxybenzoate; Methyl caffeate; 1-beta-D-Arabinofuranosyluracil; 2'-Deoxyadenosine; Cordycepin (3'-Deoxyadenosine); 4,4'-Dihydroxy-2-methoxychalcone; Echinatin; Naringenin (5,7,4'-Trihydroxyflavanone); Sophoraflavanone G; Eriodictyol (5,7,3',4'-Tetrahydroxyflavanone); Glabranine; Liquiritigenin; Naringenin-7-O-glucoside (Prunin); Choerospondin; Isobavachin; Liquiritigenin-4'-O-Glucoside (Liquiritin); Naringenin-7-O-Neohesperidoside(Naringin); Taxifolin(Dihydroquercetin); Aromadendrin (Dihydrokaempferol); Taxifolin-3-O-rhamnoside (Astilbin); Hispidulin (5,7,4'-Trihydroxy-6-methoxyflavone); Diosmetin (5,7,3'-Trihydroxy-4'-methoxyflavone); Pectolinarigenin; Wogonin (5,7-Dihydroxy-8-Methoxyflavone); Luteolin (5,7,3',4'-Tetrahydroxyflavone); Cirsimaritin (4',5-dihydroxy-6,7-dimethoxyflavone)*; Acacetin; Apigenin-7,4'-dimethyl ether; 6-Hydroxyluteolin; 4',5,7-Trihydroxy-3',6-dimethoxyflavone (Jaceosidin); 5,2'-Dihydroxy-7,8-dimethoxyflavone; Apigenin-8-C-Glucoside (Vitexin); Apigenin-7-O-glucoside(Cosmosiin); 5-Hydroxy-4',6,7-trimethoxyflavone (Salvigenin); Eupatilin (5,7-Dihydroxy-3',4',6-Trimethoxyflavone); Isorhamnetin; 3'-Methoxy-3,4',5,7-Tetrahydroxyflavone; Quercetin; Morin; Kaempferol (3,5,7,4'-Tetrahydroxyflavone); Sexangularetin; Kaempferol-7-O-glucoside; 3,5,6,7,8,3',4'-Heptamethoxyflavone; Garbanzol; Limocitrin (5,7,4'-trihydroxy-8,3'-dimethoxyflavone); Kaempferol-3-O-

Diseases		Components
		arabinoside (Juglanin); Kaempferide (3,5,7-Trihydroxy-4'-methoxyflavone); Catechin; 7-O-Methyleriodictyol; Vestitol; Astrapterocarpan; Claussequinone; Formononetin-7-O-glucoside (Ononin); Afrormosin (6,4'-Dimethoxy-7-Hydroxyisoflavone); Calycosin-7-O-glucoside; Biochanin A; 3'-Methoxydaidzein; 5,7,4'-Trihydroxy-3'-methoxyisoflavone; 3'-O-Methylorobol; Maackiain; Tectorigenin; Genistein; Glycitein; pseudobaptigenin; Irisolidone; Glycitin; 8-O-methylretusin; Genistein-7-O-Glucoside (Genistin); Pterocarpine; Formononetin (7-Hydroxy-4'-methoxyisoflavone); Daidzein-7-O-glucoside(Daidzin); Medicarpin; Trifolirhizin (Maackiain-3-O-glucoside); Sophoricoside; Daidzein; Puerarin (7,4'-Dihydroxy-8-C-glucosylisoflavone); Aloe emodin; Embelin; Epipinoresinol; Dehydrodiconiferyl alcohol; Scopoletin-7-O-glucoside (Scopolin); stevenin; Esculin (6,7-Dihydroxycoumarin-6-O-glucoside); 3,4-Dihydrocoumarin; Coumarin; Eucommiol; Capillarisin; 5,7-Dihydroxychromone; beta-D-Galactose; Stachyose; 1,5-Anhydro-D-glucitol; Nicotinamide; Pyridoxine; Riboflavin (Vitamin B2); Procyanidin B1; Procyanidin B2*; Candicine; L-Tyramine; Tryptamine; Abrine; Indole-3-acetic acid (IAA); 4-Guanidinobutyric acid; Shikimic acid; Phenylpyruvic acid; Quinic Acid; Citric Acid; Abscisic acid; Cis-Aconitic acid; Fumaric acid; L-Tartaric acid; DL-3-Phenyllactic acid*; Ricinoleic acid; 9-Hydroxy-10,12,15-octadecatrienoic acid; Punicic acid (9Z,11E,13Z-octadecatrienoic acid); γ-Linolenic Acid*; Undecylic Acid; α-Linolenic Acid*; Dodecanoic acid (Lauric acid); Methyl linolenate; Pentadecanoic Acid
	Infectious Diseases	L-Citrulline; L-Homocitrulline; Dihydroferulic Acid; Gallic acid; Hydrocinnamic acid; Salidroside; Syringaldehyde; 4-Hydroxy-3,5-Dimethoxybenzaldehyde; Sinapic acid; 3,4-Dimethoxycinnamic acid; Ferulic acid; Tyrosol; 4-Hydroxyphenylethanol; 2'-Deoxyadenosine; Cordycepin (3'-Deoxyadenosine); 4,4'-Dihydroxy-2-methoxychalcone; Echinatin; Naringenin (5,7,4'-Trihydroxyflavanone); Glabranine; Liquiritigenin; Hispidulin (5,7,4'-Trihydroxy-6-methoxyflavone); Pectolinarigenin; Wogonin (5,7-Dihydroxy-8-Methoxyflavone); Luteolin (5,7,3',4'-Tetrahydroxyflavone); Luteolin-7-o-glucuronide; Acacetin; Chrysoeriol-7-O-glucoside; Apigenin-6,8-di-C-glucoside (Vicenin-2); 4',5,7-Trihydroxy-3',6-dimethoxyflavone (Jaceosidin); 5,2'-Dihydroxy-7,8-dimethoxyflavone; Apigenin-8-C-Glucoside (Vitexin); Neoeriocitrin; 5-Hydroxy-4',6,7-trimethoxyflavone (Salvigenin); Eupatilin (5,7-Dihydroxy-3',4',6-Trimethoxyflavone); Vitexin-2''-O-rhamnoside; Quercetin-3-O-rutinoside (Rutin)*; Quercetin; Kaempferol-3-O-neohesperidoside*; Rhamnetin-3-O-rhamnoside; Kaempferol (3,5,7,4'-Tetrahydroxyflavone); Sexangularetin; Quercetin-3-O-glucoside (Isoquercitrin)*; 3,5,6,7,8,3',4'-Heptamethoxyflavone; Limocitrin (5,7,4'-trihydroxy-8,3'-dimethoxyflavone); Quercetin-3-O-(2''-O-galactosyl)glucoside; Kaempferol-3-O-glucuronide; Kaempferol-3,7-O-diglucoside; Isorhamnetin-3,7-O-diglucoside; Kaempferol-3-O-arabinoside (Juglanin); Quercetin-3-O-rhamnoside(Quercitrin); Quercetin-3-O-sophoroside (Baimaside); Kaempferide (3,5,7-Trihydroxy-4'-methoxyflavone); Catechin; Vestitol; Claussequinone; Formononetin-7-O-glucoside (Ononin); Afrormosin (6,4'-Dimethoxy-7-Hydroxyisoflavone); Biochanin A; 3'-Methoxydaidzein; 5,7,4'-Trihydroxy-3'-methoxyisoflavone; 3'-O-Methylorobol; Tectorigenin; Genistein; Isoluteolin (Orobol)(5,7,3',4'-tetrahydroxyisoflavone); Glycitein; pseudobaptigenin; Irisolidone; Glycitin; 8-O-methylretusin; Formononetin (7-Hydroxy-4'-methoxyisoflavone); Genistein-8-C-glucoside; Medicarpin; Daidzein; Syringaresinol-4'-O-glucoside; Acanthoside B; 3,4-Dihydrocoumarin; Capillarisin; 5,7-Dihydroxychromone; 5-O-Methylvisammioside; beta-D-Galactose; Icariside E5; Riboflavin (Vitamin B2); Procyanidin B1; L-Tyramine; 4-Guanidinobutyric acid; DL-Glyceric Acid; Succinic acid; Phenylpyruvic acid; Citric Acid; L-Tartaric acid; DL-3-Phenyllactic acid*

Table S2. 162 differential metabolites identified through pairwise comparison (SJvJgx vs. SJvJsc3, SJvJgx vs. SJvJsc1, SJvJgx vs. SJvJsc2)

Compounds	Q1 (Da)	Q3 (Da)	Molecular weight (Da)	Formula	Ionization model	Class	Type	KAI	PDRI
N-Acetylisatin	188.04	144.05	189.0426	C10H7NO3	[M-H]-	Alkaloids	up	-	-
3-pyridine-methanol-O-β-D-glucopyranosyl	272.11	110.06	271.1056	C12H17NO6	[M+H]+	Alkaloids	up	-	-
L-Histidine	156.08	110.07	155.0695	C6H9N3O2	[M+H]+	Amino acids and derivatives	up	-	-
L-threo-3-Methylaspartate	146.05	102.06	147.0532	C5H9NO4	[M-H]-	Amino acids and derivatives	up	-	-
Phe-HoPhe-OH	493.16	307.1	434.1477866	C24H22N2O6	[M+CH3COO]-	Amino acids and derivatives	up	-	-
Nap-Nap-OH	519.15	315.09	520.1634366	C31H24N2O6	[M-H]-	Amino acids and derivatives	up	-	-
Ser-Ile-Asn-Lys	519.28	59.01	460.2645477	C19H36N6O7	[M+CH3COO]-	Amino acids and derivatives	up	-	-
Oxiglutatione	611.15	306.07	612.152	C20H32N6O12S2	[M-H]-	Amino acids and derivatives	up	-	-
L-Methionine	150.06	61.01	149.051	C5H11NO2S	[M+H]+	Amino acids and derivatives	up	-	-
Astrapterocarpan-3-O-malonyl glucoside	549.16	301.11	548.1524	C26H28O13	[M+H]+	Flavonoids	up	-	-
Kaempferide-3-O-(6"-malonyl)glucoside	549.12	301.07	548.1166	C25H24O14	[M+H]+	Flavonoids	up	-	-
Daidzein-7-O-glucoside(Daidzin)	417.12	255.07	416.1107	C21H20O9	[M+H]+	Flavonoids	up	Yes	Yes
Medicarpin	269.08	209.06	270.0892	C16H14O4	[M-H]-	Flavonoids	up	Yes	Yes
Epicatechin glucoside*	451.12	289.07	452.1319	C21H24O11	[M-H]-	Flavonoids	up	-	-
Biochanin A-7-O-glucoside-6"-O-malonate	533.13	285.07	532.1211	C25H24O13	[M+H]+	Flavonoids	up	-	-
Medicarpin 3-O-glucoside-6'-malonate	519.15	271.1	518.1424	C25H26O12	[M+H]+	Flavonoids	up	-	-
Epicatechin-6-C-β-D-glucopyranoside*	451.13	289.07	452.1319	C21H24O11	[M-H]-	Flavonoids	up	-	-
Catechin-catechin-catechin	865.2	407.1	866.2058	C45H38O18	[M-H]-	Flavonoids	up	-	-
Daidzein-4',7-Diglucoside	579.17	255.07	578.1636	C27H30O14	[M+H]+	Flavonoids	up	-	-

Compounds	Q1 (Da)	Q3 (Da)	Molecular weight (Da)	Formula	Ionization model	Class	Type	KAI	PDRI
Riboflavin 5'-Adenosine Diphosphate	784.15	437.09	785.1571	C27H33N9O15P2	[M-H]-	Nucleotides and derivatives	up	-	-
Uridine 5'-diphosphate	402.99	158.93	404.0022	C9H14N2O12P2	[M-H]-	Nucleotides and derivatives	up	-	-
5'-Deoxy-5'-(methylthio)adenosine	298.1	136.06	297.0896	C11H15N5O3S	[M+H]+	Nucleotides and derivatives	up	-	-
Adenosine 5'-diphosphate	426.02	158.93	427.0294	C10H15N5O10P2	[M-H]-	Nucleotides and derivatives	up	-	-
Uridine-5'-Diphosphate-D-Xylose	535.04	323.03	536.0445	C14H22N2O16P2	[M-H]-	Nucleotides and derivatives	up	-	-
Uridine 5'-diphospho-D-glucose	565.05	323.03	566.055	C15H24N2O17P2	[M-H]-	Nucleotides and derivatives	up	Yes	-
Nicotinic acid adenine dinucleotide	664.12	136.06	663.1091	C21H27N7O14P2	[M+H]+	Nucleotides and derivatives	up	-	-
Methyl 2-furoate	125.02	79.02	126.0317	C6H6O3	[M-H]-	Organic acids	up	-	-
Diethyl phosphate	153.03	125	154.0395	C4H11O4P	[M-H]-	Organic acids	up	-	-
Glucose-1-phosphate*	259.02	96.97	260.0297	C6H13O9P	[M-H]-	Others	up	-	-
D-Fructose 6-Phosphate	259.02	96.97	260.0297	C6H13O9P	[M-H]-	Others	up	-	-
3-Phospho-D-glyceric acid	184.98	78.96	185.9929	C3H7O7P	[M-H]-	Others	up	-	-
8-C-glucosyl-noreugenin	355.1	235.06	354.0951	C16H18O9	[M+H]+	Others	up	-	-
D-Glucose 6-phosphate*	259.02	96.97	260.0297	C6H13O9P	[M-H]-	Others	up	-	-
Vanilloloside	315.11	153.02	316.1158	C14H20O8	[M-H]-	Phenolic acids	up	-	-
Caffeoyl(p-Hydroxybenzoyl)tartaric acid	431.06	137.03	432.0693	C20H16O11	[M-H]-	Phenolic acids	up	-	-
Salidroside	299.11	137.02	300.1209	C14H20O7	[M-H]-	Phenolic acids	up	Yes	Yes
gentisic acid 5-O-β-D-(6'-O-galloyl)-gluco-pyranoside	467.08	423.09	468.0904	C20H20O13	[M-H]-	Phenolic acids	up	-	-
Vanillic acid-4-O-glucoside	329.09	209.04	330.0951	C14H18O9	[M-H]-	Phenolic acids	up	-	-
4-O-Glucosyl-4-hydroxybenzoic acid	299.08	137.02	300.0845	C13H16O8	[M-H]-	Phenolic acids	up	-	-
Protocatechuic acid-4-O-glucoside	315.07	153.02	316.0794	C13H16O9	[M-H]-	Phenolic acids	up	-	-

Compounds	Q1 (Da)	Q3 (Da)	Molecular weight (Da)	Formula	Ionization model	Class	Type	KAI	PDRI
Protocatechuic acid 4-O-(6"-O-Feruloyl)Glucoside	491.12	315.07	492.1268	C23H24O12	[M-H]-	Phenolic acids	up	-	-
Protocatechuic acid 4-O-(6"-O-Galloy)Glucoside	467.08	315.07	468.0904	C20H20O13	[M-H]-	Phenolic acids	up	-	-
1-O-Salicyloyl-β-D-glucose	299.08	137.02	300.0845	C13H16O8	[M-H]-	Phenolic acids	up	-	-
3-O-p-Coumaroylshikimic acid	319.08	145.03	320.0896	C16H16O7	[M-H]-	Phenolic acids	up	-	-
4-(3,4,5-Trihydroxybenzoxy)benzoic acid	289.04	137.02	290.0427	C14H10O7	[M-H]-	Phenolic acids	up	-	-
Gallic acid-4-O-glucoside	331.07	313.06	332.0743	C13H16O10	[M-H]-	Phenolic acids	up	-	-
Procyanidin C1	865.2	577.14	866.2058	C45H38O18	[M-H]-	Tannins	up	-	-
Procyanidin B2*	577.14	407.08	578.1424	C30H26O12	[M-H]-	Tannins	up	-	Yes
Procyanidin B3*	577.14	407.08	578.1424	C30H26O12	[M-H]-	Tannins	up	-	-
Dendrocrepine*	575.35	279.2	516.3352	C33H44N2O3	[M+CH3COOH-H]-	Alkaloids	down	-	-
N-Glucosyl-p-coumaroylputrescine	397.2	147.04	396.1897	C19H28N2O7	[M+H]+	Alkaloids	down	-	-
L-Leucyl-L-Leucine	245.19	86.1	244.1787	C12H24N2O3	[M+H]+	Amino acids and derivatives	down	-	-
L-Valyl-L-Leucine	231.17	72.08	230.163	C11H22N2O3	[M+H]+	Amino acids and derivatives	down	-	-
Lys-Phe-Leu-Glu	534.28	452.28	535.3005989	C26H41N5O7	[M-H]-	Amino acids and derivatives	down	-	-
N-Ethylmaleimide (NEM)	126.05	80.01	125.0477	C6H7NO2	[M+H]+	Amino acids and derivatives	down	-	-
L-Aspartyl-L-Phenylalanine	281.11	166.09	280.1059	C13H16N2O5	[M+H]+	Amino acids and derivatives	down	-	-
Asn-Glu-Tyr-Glu	552.2	516.19	553.202007	C23H31N5O11	[M-H]-	Amino acids and derivatives	down	-	-
His-Ile-Lys-Arg	551.34	255.23	552.3496147	C24H44N10O5	[M-H]-	Amino acids and derivatives	down	-	-
Trp-Abu-OH	410.13	143.04	411.1430355	C21H21N3O6	[M-H]-	Amino acids and derivatives	down	-	-
Ile-Phe-Val-Lys	564.33	504.31	505.3264197	C26H43N5O5	[M+CH3COO]-	Amino acids and derivatives	down	-	-

Compounds	Q1 (Da)	Q3 (Da)	Molecular weight (Da)	Formula	Ionization model	Class	Type	KAI	PDRI
Cyclo(Phe-Glu)	277.12	120.08	276.111	C14H16N2O4	[M+H]+	Amino acids and derivatives	down	-	-
Ser-Leu	339.13	169.01	218.1266572	C9H18N2O4	[M-H]-	Amino acids and derivatives	down	-	-
L-Leucyl-L-phenylalanine	279.17	120.08	278.163	C15H22N2O3	[M+H]+	Amino acids and derivatives	down	-	-
Ac-Yvad-cho	491.21	347.17	492.2220142	C23H32N4O8	[M-H]-	Amino acids and derivatives	down	-	-
Kaempferol-3-O-(2"-galloyl)galactoside*	601.12	287.06	600.1115	C28H24O15	[M+H]+	Flavonoids	down	-	-
7-O-methylpseudobaptigenin	297.08	267.06	296.0685	C17H12O5	[M+H]+	Flavonoids	down	-	-
6-C-Methylquercetin-3-O-rhamnoside	463.12	317.06	462.1162	C22H22O11	[M+H]+	Flavonoids	down	-	-
7,4'-Di-O-galloyltricetiflavan	593.09	289.07	594.101	C29H22O14	[M-H]-	Flavonoids	down	-	-
Kaempferol-7-O-rhamnoside	431.1	285.04	432.1056	C21H20O10	[M-H]-	Flavonoids	down	Yes	-
Rhamnetin-3-O-rhamnoside	461.11	315.05	462.1162	C22H22O11	[M-H]-	Flavonoids	down	-	Yes
Apigenin-7,4'-dimethyl ether	299.09	284.07	298.0841	C17H14O5	[M+H]+	Flavonoids	down	Yes	Yes
7,3'-Di-O-gallyoltricetiflavan	593.09	289.07	594.101	C29H22O14	[M-H]-	Flavonoids	down	-	-
Kaempferol-3-O-(6"-galloyl)glucoside*	601.12	287.06	600.1115	C28H24O15	[M+H]+	Flavonoids	down	-	-
5,7,3',4',5'-Pentahydroxydihydroflavone	303.05	151.03	304.0583	C15H12O7	[M-H]-	Flavonoids	down	-	-
Puerarin (7,4'-Dihydroxy-8-C-glucosylisoflavone)	417.12	297.1	416.1107	C21H20O9	[M+H]+	Flavonoids	down	Yes	Yes
Isohydroxymatairesinol	373.13	329.05	374.1366	C20H22O7	[M-H]-	Lignans and Coumarins	down	-	-
LysoPC 18:3	518.32	184.07	517.3168	C26H48NO7P	[M+H]+	Lipids	down	-	-
LysoPC 16:0	496.34	184.07	495.3325	C24H50NO7P	[M+H]+	Lipids	down	-	-
2-α-Linolenoyl-glycerol*	353.27	261.22	352.2614	C21H36O4	[M+H]+	Lipids	down	-	-
LysoPC 18:3(2n isomer)	518.32	184.07	517.3168	C26H48NO7P	[M+H]+	Lipids	down	-	-
12,13-DHOME; (9Z)-12,13-Dihydroxyoctadec-9-enoic acid	313.24	251.24	314.2457	C18H34O4	[M-H]-	Lipids	down	-	-

Compounds	Q1 (Da)	Q3 (Da)	Molecular weight (Da)	Formula	Ionization model	Class	Type	KAI	PDRI
LysoPE 18:0(2n isomer)	482.32	341.31	481.3168	C23H48NO7P	[M+H]+	Lipids	down	-	-
LysoPE 18:2	478.29	337.27	477.2855	C23H44NO7P	[M+H]+	Lipids	down	-	-
LysoPE 17:0	468.31	327.29	467.3012	C22H46NO7P	[M+H]+	Lipids	down	-	-
LysoPC 18:1	522.36	184.07	521.3481	C26H52NO7P	[M+H]+	Lipids	down	-	-
LysoPC 17:0(2n isomer)	510.36	184.07	509.3481	C25H52NO7P	[M+H]+	Lipids	down	-	-
LysoPC 16:2(2n isomer)	492.31	184.07	491.3012	C24H46NO7P	[M+H]+	Lipids	down	-	-
LysoPE 18:1	480.31	339.29	479.3012	C23H46NO7P	[M+H]+	Lipids	down	-	-
Punicic acid (9Z,11E,13Z-octadecatrienoic acid)	279.23	95.09	278.2246	C18H30O2	[M+H]+	Lipids	down	Yes	Yes
LysoPC 20:3	546.36	184.07	545.3481	C28H52NO7P	[M+H]+	Lipids	down	-	-
LysoPE 15:0	440.28	299.26	439.2699	C20H42NO7P	[M+H]+	Lipids	down	-	-
13S-Hydroxy-9Z,11E,15Z-octadecatrienoic acid	293.21	195.14	294.2195	C18H30O3	[M-H]-	Lipids	down	-	-
1-(2,3-dihydroxypropoxy)-3-(((2-(dimethylamino)ethoxy)(hydroxy)phosphoryl)oxy)propan-2-yl palmitate	554.35	255.23	555.3536	C26H54NO9P	[M-H]-	Lipids	down	-	-
LysoPC 18:0(2n isomer)	524.37	184.07	523.3638	C26H54NO7P	[M+H]+	Lipids	down	-	-
2-Palmitoyl-Sn-Glycerol 3-O-Diglucoside	653.38	397.14	654.3827	C31H58O14	[M-H]-	Lipids	down	-	-
Hexadecanedioic acid	285.21	223.21	286.2144	C16H30O4	[M-H]-	Lipids	down	Yes	-
2-(2,3-dihydroxypropoxy)-3-(((2-(dimethylamino)ethoxy)(hydroxy)phosphoryl)oxy)propyl (11Z,14Z)-octadeca-11,14-dienoate*	578.34	279.23	579.3536	C28H54NO9P	[M-H]-	Lipids	down	-	-
1-(9Z-octadecenoyl)-sn-glycero-3-phosphocholine	580.36	506.33	521.3481402	C26H52NO7P	[M+CH3COO]-	Lipids	down	-	-
LysoPC 16:0(2n isomer)	496.34	184.07	495.3325	C24H50NO7P	[M+H]+	Lipids	down	-	-
LysoPE 16:0	454.29	313.27	453.2855	C21H44NO7P	[M+H]+	Lipids	down	-	-
LysoPC 18:0	524.37	184.07	523.3638	C26H54NO7P	[M+H]+	Lipids	down	-	-
LysoPC(18:3(9Z,12Z,15Z))	576.33	502.3	517.3168401	C26H48NO7P	[M+CH3COO]-	Lipids	down	-	-

Compounds	Q1 (Da)	Q3 (Da)	Molecular weight (Da)	Formula	Ionization model	Class	Type	KAI	PDRI
LysoPE 15:0(2n isomer)	440.28	299.26	439.2699	C20H42NO7P	[M+H]+	Lipids	down	-	-
LysoPC 17:2	506.32	184.07	505.3168	C25H48NO7P	[M+H]+	Lipids	down	-	-
LysoPC 20:1	550.39	184.07	549.3794	C28H56NO7P	[M+H]+	Lipids	down	-	-
LysoPC 20:0	552.4	184.07	551.3951	C28H58NO7P	[M+H]+	Lipids	down	-	-
1-α-Linolenoyl-glycerol*	515.32	261.22	514.3142	C27H46O9	[M+H]+	Lipids	down	-	-
LysoPC 20:2(2n isomer)	548.37	184.07	547.3638	C28H54NO7P	[M+H]+	Lipids	down	-	-
LysoPC 20:2	548.37	184.07	547.3638	C28H54NO7P	[M+H]+	Lipids	down	-	-
LysoPE 20:2(2n isomer)	506.32	365.31	505.3168	C25H48NO7P	[M+H]+	Lipids	down	-	-
LysoPC 16:2	492.31	184.07	491.3012	C24H46NO7P	[M+H]+	Lipids	down	-	-
LysoPE 20:3	504.31	363.29	503.3012	C25H46NO7P	[M+H]+	Lipids	down	-	-
2-Linoleoylglycerol*	355.28	263.24	354.277	C21H38O4	[M+H]+	Lipids	down	-	-
LysoPC 16:1	494.32	184.07	493.3168	C24H48NO7P	[M+H]+	Lipids	down	-	-
LysoPC 15:0(2n isomer)	482.32	184.07	481.3168	C23H48NO7P	[M+H]+	Lipids	down	-	-
1-(2,3-dihydroxypropoxy)-3-(((2-(dimethylamino)ethoxy)(hydroxy)phosphoryl)oxy)propan-2-yl (8E,11Z,14Z)-octadeca-8,11,14-trienoate*	576.33	277.22	577.338	C28H52NO9P	[M-H]-	Lipids	down	-	-
LysoPE 20:3(2n isomer)	504.31	363.29	503.3012	C25H46NO7P	[M+H]+	Lipids	down	-	-
LysoPC 18:2(2n isomer)	520.34	184.07	519.3325	C26H50NO7P	[M+H]+	Lipids	down	-	-
2-(2,3-dihydroxypropoxy)-3-(((2-(dimethylamino)ethoxy)(hydroxy)phosphoryl)oxy)propyl (8E,11Z,14Z)-octadeca-8,11,14-trienoate*	576.33	277.22	577.338	C28H52NO9P	[M-H]-	Lipids	down	-	-
LysoPE 20:2	506.32	365.31	505.3168	C25H48NO7P	[M+H]+	Lipids	down	-	-
LysoPE 18:0	482.32	341.31	481.3168	C23H48NO7P	[M+H]+	Lipids	down	-	-
Gingerglycolipid B	677.38	397.13	678.3827	C33H58O14	[M-H]-	Lipids	down	Yes	-

Compounds	Q1 (Da)	Q3 (Da)	Molecular weight (Da)	Formula	Ionization model	Class	Type	KAI	PDRI
2-Linoleoylglycerol-1,3-di-O-glucoside*	679.39	263.24	678.3827	C33H58O14	[M+H]+	Lipids	down	-	-
1-beta-D-Arabinofuranosyluracil	245.07	113.03	244.0695	C9H12N2O6	[M+H]+	Nucleotides and derivatives	down	-	Yes
5-Methyl-2'-Deoxycytidine	242.11	126.07	241.1063	C10H15N3O4	[M+H]+	Nucleotides and derivatives	down	-	-
Cytidine	244.09	112.05	243.0855	C9H13N3O5	[M+H]+	Nucleotides and derivatives	down	-	-
2'-Deoxyadenosine	252.11	136.06	251.1018	C10H13N5O3	[M+H]+	Nucleotides and derivatives	down	Yes	Yes
Uridine	243.06	110.02	244.0695	C9H12N2O6	[M-H]-	Nucleotides and derivatives	down	-	-
Cytosine	112.05	95.02	111.0433	C4H5N3O	[M+H]+	Nucleotides and derivatives	down	-	-
2'-Deoxyinosine	253.09	137.05	252.0859	C10H12N4O4	[M+H]+	Nucleotides and derivatives	down	-	-
3'-Adenylic Acid	346.06	211	347.0631	C10H14N5O7P	[M-H]-	Nucleotides and derivatives	down	-	-
Guanosine	284.1	152.06	283.0917	C10H13N5O5	[M+H]+	Nucleotides and derivatives	down	Yes	-
Cordycepin (3'-Deoxyadenosine)	252.11	136.06	251.1018	C10H13N5O3	[M+H]+	Nucleotides and derivatives	down	Yes	Yes
Cytarabine	244.09	112.05	243.0855	C9H13N3O5	[M+H]+	Nucleotides and derivatives	down	-	-
Crotonoside; 2-Hydroxyadenosine	284.1	152.06	283.0917	C10H13N5O5	[M+H]+	Nucleotides and derivatives	down	-	-
2'-Deoxycytidine	228.1	112.05	227.0906	C9H13N3O4	[M+H]+	Nucleotides and derivatives	down	-	-
N7-Methylguanosine	298.11	166.07	297.1073	C11H15N5O5	[M+H]+	Nucleotides and derivatives	down	-	-
Thymidine	243.1	127.05	242.0903	C10H14N2O5	[M+H]+	Nucleotides and derivatives	down	-	-
N6-methyladenosine	282.12	150.08	281.1124	C11H15N5O4	[M+H]+	Nucleotides and derivatives	down	-	-

Compounds	Q1 (Da)	Q3 (Da)	Molecular weight (Da)	Formula	Ionization model	Class	Type	KAI	PDRI
8-Hydroxyguanosine	298.08	208.05	299.0866	C10H13N5O6	[M-H]-	Nucleotides and derivatives	down	-	-
Cytidine 5'-monophosphate(Cytidylic acid)	324.06	112.05	323.0519	C9H14N3O8P	[M+H]+	Nucleotides and derivatives	down	-	-
Guanosine 5'-monophosphate	364.07	152.06	363.058	C10H14N5O8P	[M+H]+	Nucleotides and derivatives	down	-	-
Uridine 5'-monophosphate	323.03	211	324.0359	C9H13N2O9P	[M-H]-	Nucleotides and derivatives	down	Yes	-
γ-Aminobutyric acid	104.07	69.03	103.0633	C4H9NO2	[M+H]+	Organic acids	down	-	-
2-Picolinic acid	122.02	78.03	123.032	C6H5NO2	[M-H]-	Organic acids	down	-	-
2-Hydroxy-4-methylpentanoic acid	131.07	85.07	132.0786	C6H12O3	[M-H]-	Organic acids	down	-	-
Glucopyranose 6-Hydroxydecanoate	409.21	161.05	350.1941	C16H30O8	[M+CH3COOH-H]-	Others	down	-	-
Isonicotinic acid	124.04	78.03	123.032	C6H5NO2	[M+H]+	Others	down	-	-
Nicotinic acid (Vitamin B3)	124.04	78.03	123.032	C6H5NO2	[M+H]+	Others	down	-	-
Glucosyl 3,7-dimethyloct-2-ene-1,6,7-triol	409.21	187.13	350.1941	C16H30O8	[M+CH3COOH-H]-	Others	down	-	-
Dihydroferulic Acid	195.07	136.05	196.0736	C10H12O4	[M-H]-	Phenolic acids	down	-	Yes
4-O-Methylgallic Acid	183.03	124.02	184.0372	C8H8O5	[M-H]-	Phenolic acids	down	-	-
3-O-Methylgallic Acid	183.03	124.02	184.0372	C8H8O5	[M-H]-	Phenolic acids	down	-	-
Methyl gallate	183.03	124.02	184.0372	C8H8O5	[M-H]-	Phenolic acids	down	-	Yes
p-Dimeric galloyl methyl ester	335.04	183.03	336.0481	C15H12O9	[M-H]-	Phenolic acids	down	-	-
1,2,3-Tri-O-galloyl-β-D-glucose	635.09	169.01	636.0963	C27H24O18	[M-H]-	Phenolic acids	down	-	-
Digallic Acid	321.03	125.02	322.0325	C14H10O9	[M-H]-	Phenolic acids	down	Yes	Yes
Gallic acid	169.01	125.02	170.0215	C7H6O5	[M-H]-	Phenolic acids	down	-	Yes
L-Malic acid-2-O-gallate	285.03	133.02	286.0325	C11H10O9	[M-H]-	Phenolic acids	down	-	-
3-O-Feruloylquinic acid	369.12	177.05	368.1107	C17H20O9	[M+H]+	Phenolic acids	down	-	-
Gallic acid-1-O-xyloside	301.06	168.01	302.0638	C12H14O9	[M-H]-	Tannins	down	-	-
uralsaponin S	971.48	469.33	970.4773	C48H74O20	[M+H]+	Terpenoids	down	-	-

Table S3. KEGG annotation and pathway mapping for 39 KEGG-annotated metabolites of 162 shared differential metabolites in all comparisons between the FBSJvJ groups (20 up-regulated and 19 down-regulated)

**Compounds**	**Level**	**KAI**	**PDRI**	**Type**	**KEGG_map**
L-Histidine	3	-	-	up	ko00340, ko00410, ko00470, ko00970, ko01100, ko01110, ko01230, ko02010
Riboflavin 5'-Adenosine Diphosphate	3	-	-	up	ko00740, ko01100, ko01110, ko01240
L-threo-3-Methylaspartate	1	-	-	up	ko00630, ko00660, ko01100, ko01200
Uridine 5'-diphosphate	3	-	-	up	ko00240, ko00908, ko01100, ko01232, ko01240
5'-Deoxy-5'-(methylthio)adenosine	1	-	-	up	ko00270, ko00908, ko01100
N-Acetylisatin	1	-	-	up	ko00380
Adenosine 5'-diphosphate	3	-	-	up	ko00190, ko00195, ko00230, ko00908, ko01100, ko01110, ko01232, ko01240
Salidroside	1	-	-	up	ko00350, ko01100
Daidzein-7-O-glucoside(Daidzin)	1	-	-	up	ko00943, ko01100
Glucose-1-phosphate*	1	-	-	up	ko00010, ko00040, ko00052, ko00500, ko00520, ko00523, ko00561, ko01100, ko01110, ko01240, ko01250
Uridine-5'-Diphosphate-D-Xylose	2	-	-	up	ko00520, ko00908, ko01100, ko01250
D-Fructose 6-Phosphate	3	-	-	up	ko00010, ko00030, ko00051, ko00052, ko00500, ko00520, ko00710, ko01100, ko01110, ko01200, ko01230, ko01240, ko01250
Medicarpin	1	-	-	up	ko00943, ko01100, ko01110
Medicarpin 3-O-glucoside-6'-malonate	1	Yes	Yes	up	**ko00943**
3-Phospho-D-glyceric acid	2	-	-	up	ko00010, ko00030, ko00260, ko00270, ko00561, ko00630, ko00710, ko01100, ko01110, ko01200, ko01230, ko01240
Uridine 5'-diphospho-D-glucose	1	Yes	-	up	ko00040, ko00052, ko00053, ko00500, ko00520, ko00524, ko00561, ko00908, ko01100, ko01110, ko01240, ko01250
Nicotinic acid adenine dinucleotide	3	-	-	up	ko00190, ko00730, ko00760, ko01100, ko01240, ko04148
D-Glucose 6-phosphate*	1	-	-	up	ko00500, ko00524, ko00562, ko01100, ko01110
Oxiglutatione	3	-	-	up	ko00480, ko01100, ko01240

Compounds	Level	KAI	PDRI	Type	KEGG_map
L-Methionine	3	-	-	up	ko00270, ko00470, ko00966, ko00970, ko00999, ko01100, ko01110, ko01210, ko01230, ko01240, ko04148, ko04980
Cytidine	2	-	-	down	ko00240, ko01100, ko01232, ko02010
2'-Deoxyadenosine	1	-	-	down	ko00230, ko01100, ko01232, ko02010
12,13-DHOME; (9Z)-12,13-Dihydroxyoctadec-9-enoic acid	2	-	-	down	ko00591
Uridine	1	-	-	down	ko00240, ko01100, ko01232, ko02010
Cytosine	3	-	-	down	ko00240, ko01100, ko01232
2'-Deoxyinosine	3	-	-	down	ko00230, ko01100, ko01232, ko02010
γ-Aminobutyric acid	3	-	-	down	ko00250, ko00330, ko00410, ko00650, ko00760, ko01100
3'-Adenylic Acid	3	-	-	down	ko00230, ko01100
Guanosine	1	-	-	down	ko00230, ko01100, ko01232, ko02010
13S-Hydroxy-9Z,11E,15Z-octadecatrienoic acid	2	-	-	down	ko00592
Hexadecanedioic acid	1	-	-	down	ko00073, ko01100
2'-Deoxycytidine	3	-	-	down	ko00240, ko01100, ko01232, ko02010
Gallic acid	1	-	-	down	ko00999
Thymidine	3	-	-	down	ko00240, ko01100, ko01232
2-Picolinic acid	3	-	-	down	ko00380, ko01100
Nicotinic acid (Vitamin B3)	3	-	-	down	ko00760, ko00960, ko01100, ko01110, ko01240
Cytidine 5'-monophosphate (Cytidylic acid)	3	-	-	down	ko00240, ko01100, ko01232
Guanosine 5'-monophosphate	3	-	-	down	ko00230, ko01100, ko01232
Uridine 5'-monophosphate	1	-	-	down	ko00240, ko01100, ko01232, ko01240

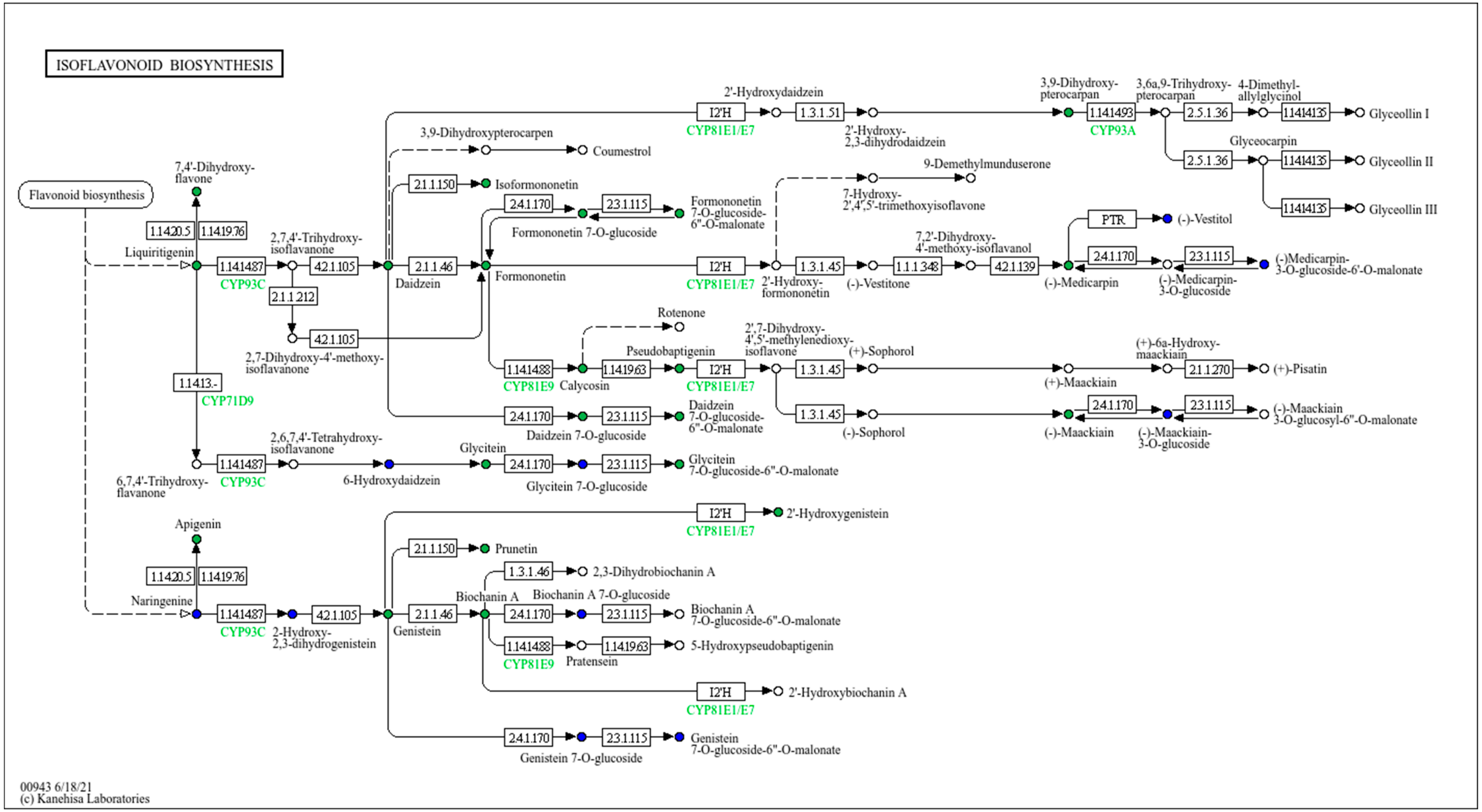

Figure S1. Differential metabolites KEGG pathway map of isoflavonoid biosynthesis (ko00943) in SJsd vs. SJah.

Note: Blue indicates detected metabolites with no significant change, and green indicates metabolites significantly downregulated in the SJah group.

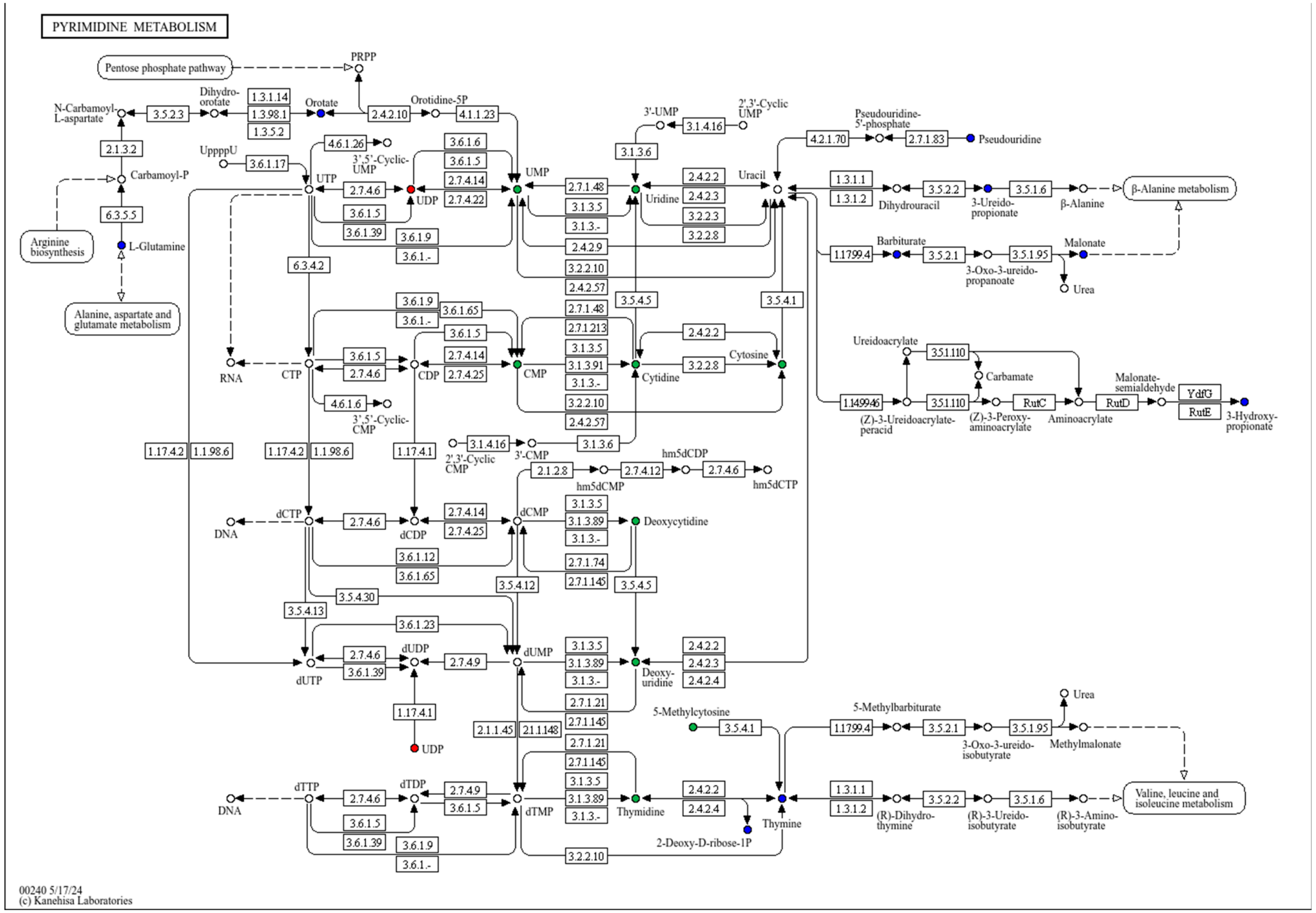

Figure S2. KEGG pathway graph of differential metabolites from pyrimidine metabolism when comparing SJvJgx with SJvJsc1(ko00240).

Note: Red indicates metabolites significantly upregulated in the SJvJsc1 group, blue indicates detected metabolites with no significant change, and green indicates metabolites significantly downregulated in the SJvJsc1 group.

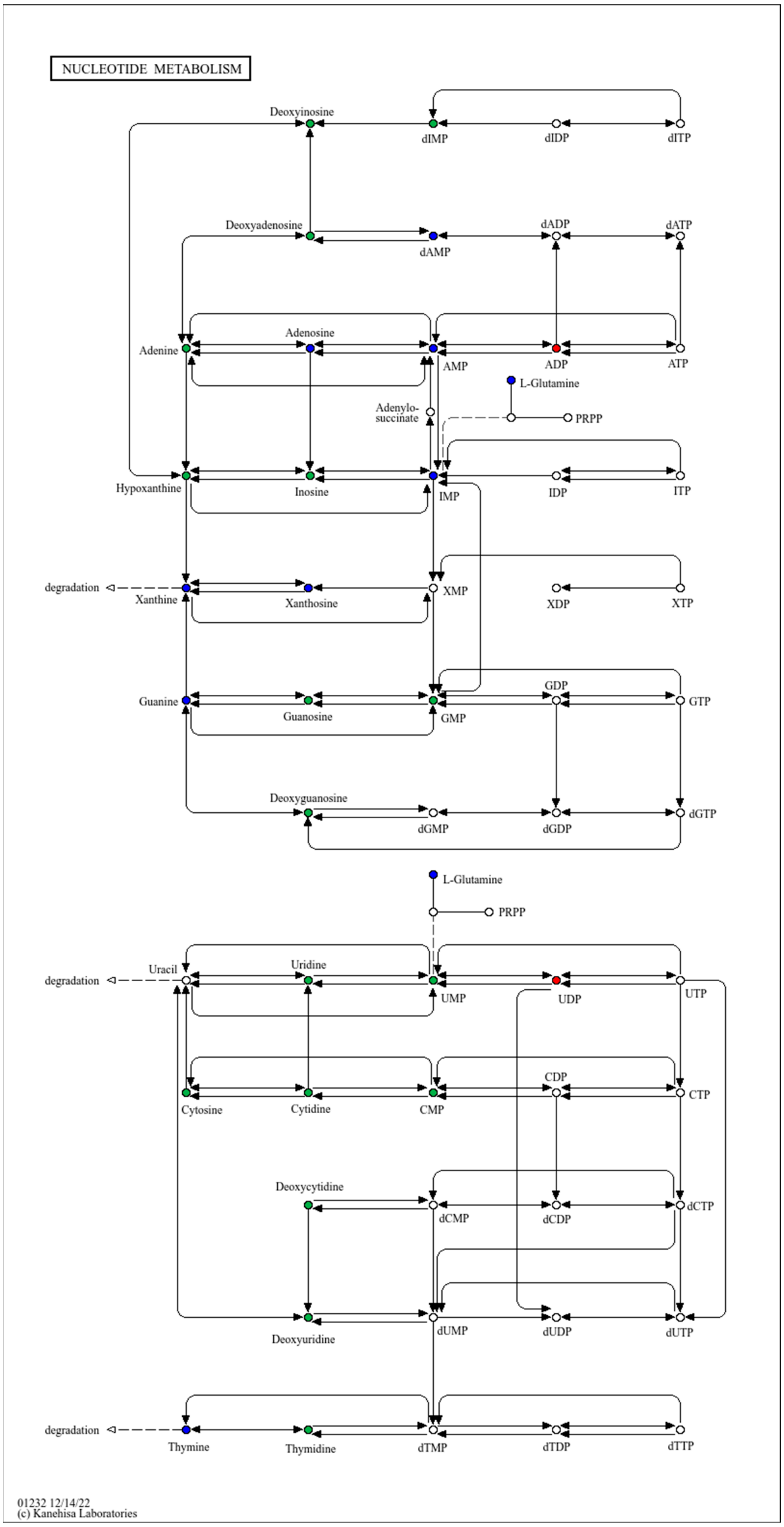

Figure S3. KEGG pathway graph of differential metabolites from nucleotide metabolism (ko01232) when comparing SJvJgx with SJvJsc1

Note: SJvJsc1.Red indicates metabolites significantly upregulated in the SJvJsc1 group, blue indicates detected metabolites with no significant change, and green indicates metabolites significantly downregulated in the SJvJsc1 group.
